# Investigating expectations and needs regarding the use of large language models at Bavarian university clinics

**DOI:** 10.1038/s41598-026-45245-2

**Published:** 2026-03-26

**Authors:** Juraj Vladika, Alexander Fichtl, Florian Matthes

**Affiliations:** Department of Computer Science, TUM School of Computation, Information, and Technology, Technical University of Munich, Garching, Germany

**Keywords:** Large language models, Artificial intelligence, Natural language processing, Medical AI, Digital transformation, Computer science, Information technology, Medical research, Health care, Health policy

## Abstract

Recent advancements in Artificial Intelligence (AI) have been driven by Large Language Models (LLMs), powerful tools capable of generating coherent text and solving diverse analytical tasks. While LLMs hold great potential to enhance healthcare by assisting physicians and improving patient treatment, their clinical adoption is limited, and there is a lack of statistically grounded information on the opinions of medical professionals, personnel, and students regarding LLM usage. To address this gap, we conducted an online survey from April to October 2024, gathering insights from 120 participants across five Bavarian university clinics (in Germany), including physicians, medical students, and administrative staff. Findings show that many participants already use LLMs for research support, summarization, translation, and report drafting. Most believe LLMs will positively influence their field, acknowledge their potential to automate mundane tasks, and believe they will help to achieve a more personalized, evidence-based, and cost-effective patient treatment. However, concerns were shown regarding their opaque nature, data privacy, and the potential loss of patient trust. Participants overwhelmingly feel their institutions are not well-prepared for LLM adoption, with suggestions for improvement including increased education and specialized training, investments in digitalization and infrastructure, ensuring legal compliance, and encouraging technological openness. We hope these insights will inform the design of future medical AI solutions.

## Introduction

The field of Artificial Intelligence (AI) focuses on building machines that can solve tasks requiring advanced analytical skills and human-level intelligence^1^. At the intersection of AI and linguistics stands Natural Language Processing (NLP), a discipline dealing with developing tools that can analyze and understand human-written text or generate new text from scratch^2^. Impressive progress in NLP in the last few years has been achieved due to the advent of Large Language Models (LLMs), powerful tools that can generate text and solve a wide array of tasks using their next-word prediction capabilities based on the transformer architecture and pre-training on massive textual corpora^3^. Along with popular general-purpose LLMs like ChatGPT^4^, models specifically trained for biomedical purposes have also emerged^5^.

LLMs have shown high capabilities in assisting with diverse and complex medical tasks and a promising potential in improving patient treatment and hospital systems^6^. This includes generation of discharge summaries^7^, empathetic conversations with patients^8^, encoding clinical knowledge for answering medical questions^9,10^, or assisting medical students with preparations for entry exams^11^. Conversely, other studies revealed that LLMs can still struggle with accurate patient diagnosis, adhering to clinical guidelines, and being applied in real hospital settings^12^. Nevertheless, as they have already started transforming numerous parts of society, the quick pace of their improvement makes a broad inclusion of LLMs into hospital systems highly likely in upcoming years^13^. Therefore, it is of crucial importance to understand the implications of LLMs in medicine. Studies investigating the potential of LLMs in other high-stake fields like law^14^, science^15^, and education^16^, have started emerging. In the medical field, however, previous studies focused mostly on AI models working with numerical or visual data instead of natural language^17,18^. Initial studies discussing LLMs in medical work have started appearing^19,20^, but there is still a lack of comprehensive surveys on the opinions of clinicians and medical students on the use of LLMs in their work.

While the United States and China lead the current AI revolution, Germany also ranks among the top contributors to AI research output^21^. However, a sharp contrast exists between this high research productivity and the real-world practical implementation of AI solutions. Germany is marked by significantly slower adoption of these technologies in the public sector, largely due to stricter legal regulations and a culture that prioritizes established practices over potentially risky innovations^22^. The legal regulations governing medical AI include the EU’s General Data Protection Regulation (GDPR)^23^, focusing on individuals’ rights to data protection and privacy including patient data, and Medical Device Regulation^24^, which addresses safety, quality, and risk management requirements on all medical technology of the market. The level of digitalization in German hospital systems is still rather modest^25^, including the low adoption of AI in healthcare^26^, and before introducing tools based on LLMs into the German medical sector, it is essential to understand how clinicians currently perceive these technologies. By understanding what medical professionals and students prioritize or are concerned about, developers and researchers can tailor biomedical NLP solutions to better fit the actual needs of the medical community.

To help bridge this research gap, we conducted a quantitative study using a survey instrument which also included qualitative elements. The survey consists of 22 questions and targets three groups: (a) physicians, (b) students of medicine, and (c) hospital administrative staff. The survey inquires about the opinions and attitudes of these three groups on the use of medical LLMs in their daily work. The questions include asking about potential use cases they would like to use the LLMs for, opinions on how it will affect the workforce, evaluating the acceptable level of errors, inquiry about what the perceived benefits are but also the risks and dangers of such models. A total of 120 participants completed the survey (70 students, 36 physicians, 14 administrative) from 5 German (Bavarian) university clinics between April and October 2024. We hope the results of the survey will help bridge the gap between the development of LLM-based solutions and their actual adoption in applied clinical settings by medical professionals.

## Results

In this section, we present the results of the research survey. We discuss the survey questions within their respective thematic groups. We present the most informative insights, while complete survey results are included in the Supplementary materials.

### Participant background

In total, 120 complete questionnaire responses were received across the three target demographic groups. Their distribution was as follows:

- Ongoing studies in the field of medicine: 70 participants
- Working as a physician: 36 participants
- Administrative staff: 14 participants

The physicians and resident students were specialized in various fields, including psychiatry (13), neurology (6), general practitioners (4), internal medicine (3), otorhinolaryngology (ENT) (3), orthopedic surgery (3), radiology (2), nephrology (2), urology (2), translational medicine (2) and a few others. The full list can be found in the Supplementary materials. The sample is slightly biased towards psychiatry and neurology because these departments had the most active distribution of our survey.

Out of 80 participants that responded about their years of experience in medicine, 20 (25%) have worked below 5 years, 18 (22.5%) have worked 5–10 years, 13 (16.25%) have worked 10–20 years, 7 (8.75%) have worked 20–30 years, and 2 (2.5%) have worked more than 30 years.

### Current knowledge and use of NLP

**Fig. 1:**
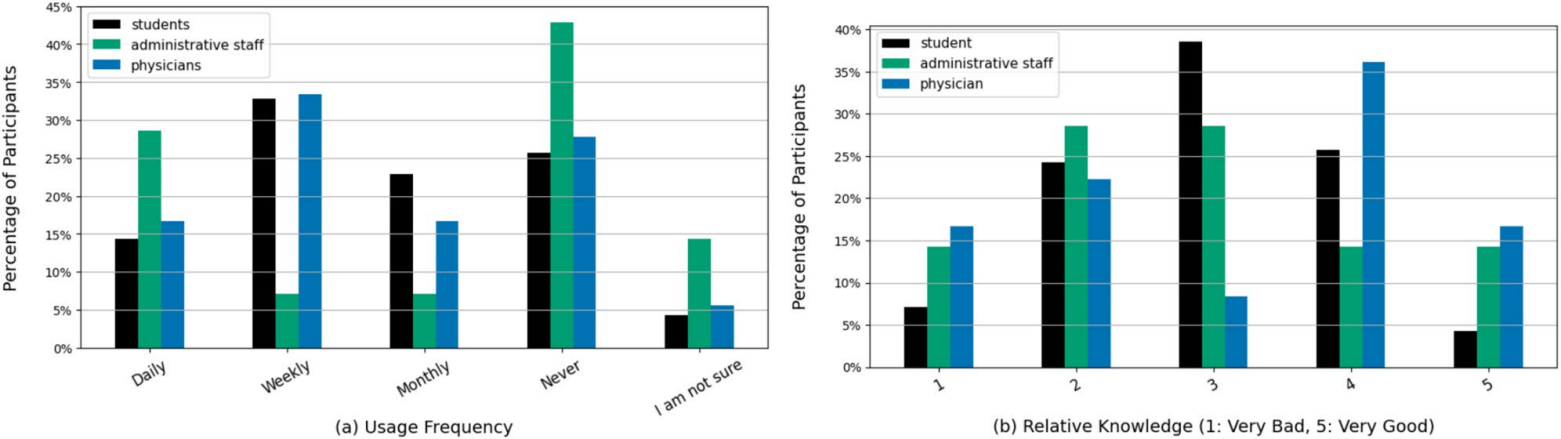
Left plot (**a**):“How often do you use LLMs in your daily work?”Right plot (**b**):“Compared to your colleagues, how would you rate your knowledge of LLMs (on a scale of 1 to 5)?”.

While 34 participants (28.33%) never used LLMs for work-related tasks, almost half of the participants (n=56; 46.67%) reported a daily or weekly use (Fig. 1). Around one quarter of students (n=18; 25.7%) and physicians (n=10; 27.8%) indicated that they never use LLMs, while around one half of students (n=33; 47.1%) and exactly one half of physicians (n=18; 50%) said they use LLMs on a weekly or daily basis. Administrative staff showed the lowest frequency of use, with 6 (33.3%) reporting never using LLMs and only 5 (27.8%) using it weekly or daily. Still, the usage between groups is relatively similar (p=0.335, V=0.195).

The participants’ confidence in their knowledge of LLMs varied significantly between groups (p = 0.037). While the largest chunk of students (n=27, 38.57%) considered their knowledge average (score of 3), physicians were very confident in their knowledge. The majority of physicians rated their knowledge of LLMs as good (4) or very good (5) (n=19, 52.78%, compared to only n=21, 30.00% of students). The responses of the administrative staff were less conclusive. When asked how they would rate their knowledge of LLMs compared to their colleagues, 42 participants (35.00%) reported that they feel they have a worse understanding of this technology than their colleagues.

The participants mentioned a wide range of use cases for which they already utilize LLMs. The most popular use case was using LLMs as a general support tool for doing research (data analysis, literature search, research ideas, etc.), mentioned 17 times. Other popular use cases (with number of mentions) were using LLMs for answering questions (14), translation (11), explaining unfamiliar concepts (10), and general help with studying (5); various types of text generation such as drafting of e-mails (7), patient discharge notes (*Arztbriefe*) (5), and parts of research work like master’s thesis and scientific articles (8). Other mentioned use cases include summarization (6), programming (4), spellchecking (4), and speech transcription (3).

Other than inquiring about their existing use cases, we also prepared a pre-defined set of popular practical use cases of LLMs and asked participants about the relevance for their daily work, even if they do not use them yet. Some of the proposed use cases overlap with those already found in the free-form field: translation, report creation, summarization, and question answering. Still, some of the use cases included in this question were rarely mentioned in the free-form answers: information extraction, speech transcription, and text simplification.

**Fig. 2:**
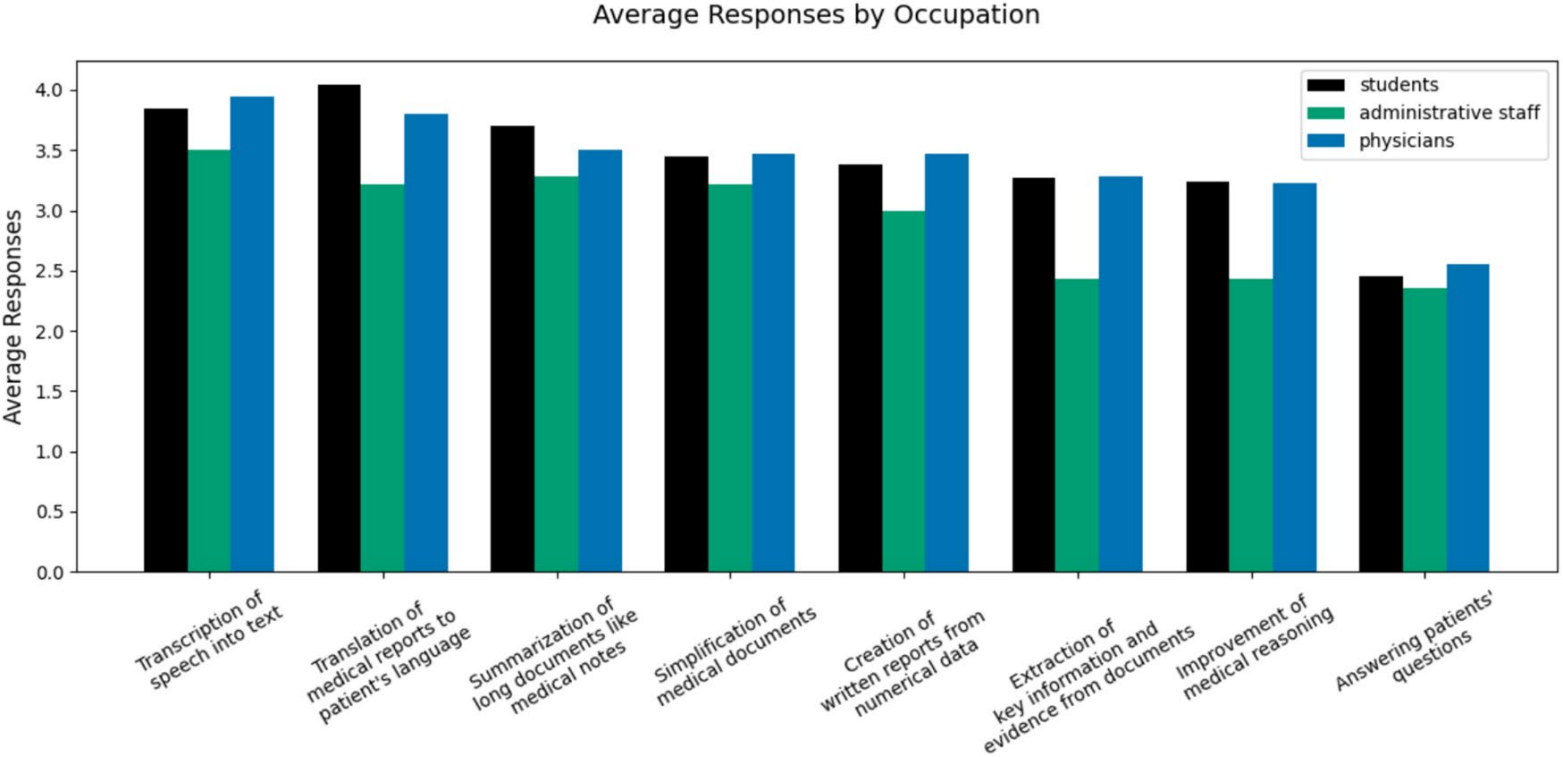
“How relevant are the following use cases of language models to your daily work? (not only for your current use but also for potential future use)?”(on a scale from 1 to 5).

The average results of relevance (scale 1–5) of potential LLM use cases in their work are in Fig. 2. Each potential use case was rated on average above 2.35. The use cases seen as the most relevant across all groups were translating medical reports (4.04, 3.21, 3.81) and leveraging LLMs for transcribing spoken language into text (3.84, 3.50, 3.94). These two use cases were then closely followed by the three generative tasks, namely report creation, text summarization, and text simplification. The administrative staff considered the summarizing of long documents slightly more important than translation, and in general rated medical-specific tasks considerably lower than physicians and students did. Using LLMs for key information extraction and for providing medical reasoning were also relatively popular among physicians and students (average above 3), but less relevant for the administrative stuff. Finally, the least attractive use case among all three participant groups was answering patient questions during their daily work (2.46, 2.36, 2.56).

### Perceived influence

More than one-third of all respondents (n=26, 38.24%) already perceive a noticeable impact of LLMs on their field of practice today. The second largest group of respondents (n=23; 33.82%) estimates that an impact will be evident within 5-10 years. Only two participants (2.94%) expect that it will take more than 10 years for an impact to be felt in their field. Notably, no medical students were among them, even though it is by far the largest participant group. The three groups responded similarly (p=0.175, V = 0.22).

**Fig. 3:**
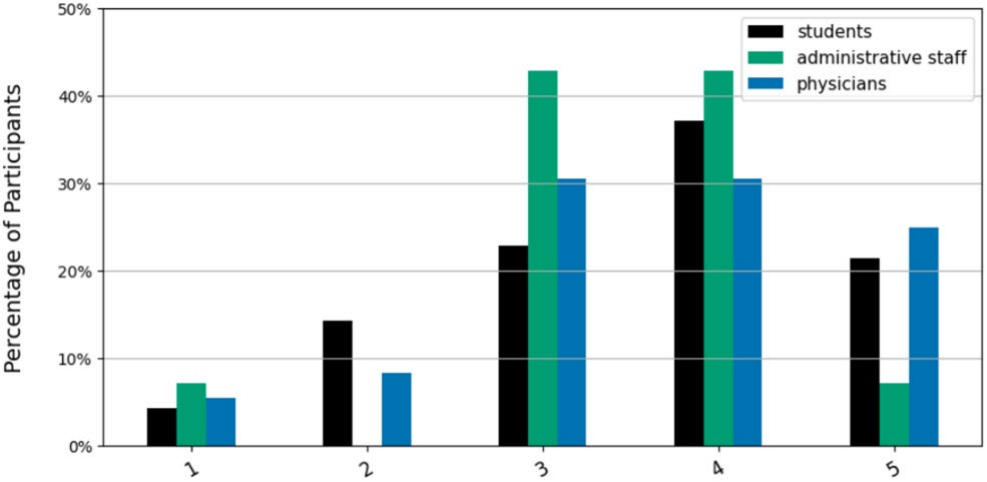
“How much do you agree with the following statement: * The introduction of LLMs will improve my field* (1: Completely disagree; 5: Completely agree).”.

When asked about the nature of the expected or present impact (Fig. 3), the vast majority of participants anticipated that the integration of LLMs would improve their current or future professional domain (n=68; 56.67% agreed or strongly agreed). Notably, while students and physicians gave similar responses, the administrative staff was less enthusiastic, with most responses (n=6; 42.86%) corresponding to a neutral expectation.

**Fig. 4:**
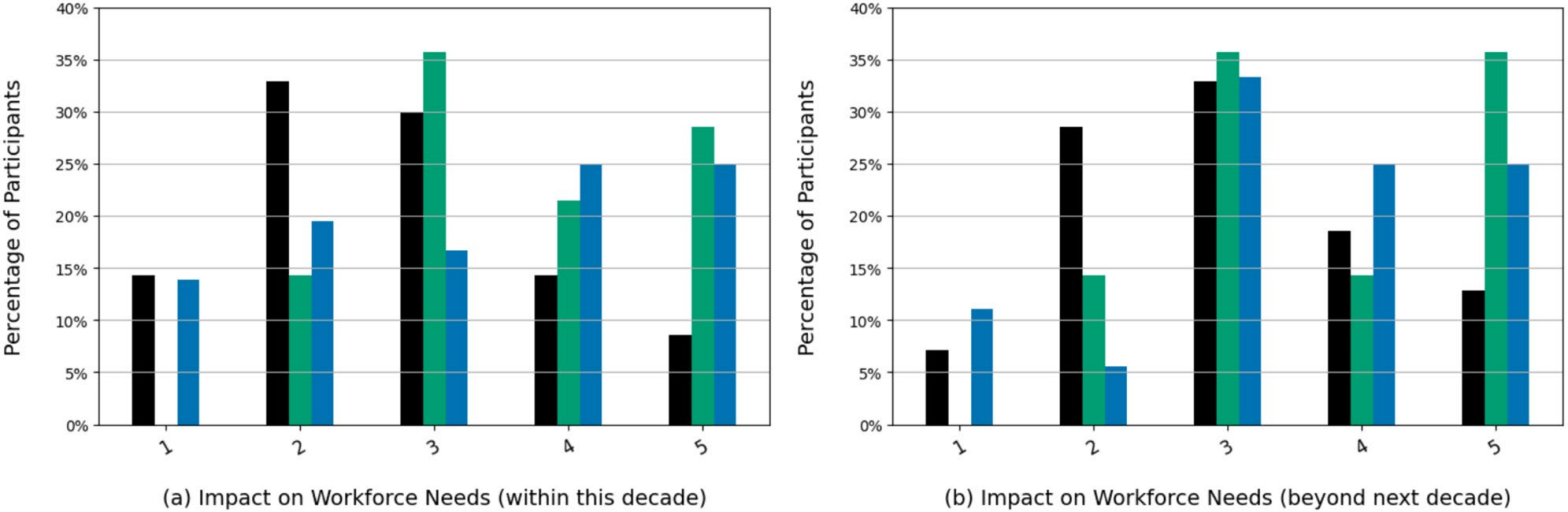
“To what extent will language models have an impact on workforce needs in your area of expertise within the next decade (**a**) versus beyond the next decade (**b**)? (on a scale from 1=low impact to 5=high impact)”.

Figure 4 focuses on the perception regarding changes in workforce needs. While (n=41; 34.17%) expect a strong or very strong change in workforce needs due to LMs within this decade, (n=47; 39.17%) disagree and only predict small or very small changes. These numbers shift slightly when looking beyond the next decade, where only (n=33; 27.5%) still disagree or strongly disagree. Overall, students expect the workforce needs to change less so than physicians and administrative staff within this decade (p=0.025, $\eta ^2$=0.448) and beyond the next decade (p=0.005, $\eta ^2$=0.733)

While the previous two questions focused on the strength of impact on workforce needs, the next question asked about the type of change (increase, decrease, remain the same). Hereby, the participants in our survey indicated that they anticipate a decrease in workforce needs (n=52; 43.33%) much more strongly than an increase (n=7; 5.83%). Surprisingly, with this phrasing, the largest share of participants (n=61; 50.83%) anticipates that the workforce needs will neither increase nor decrease.

### Practical implications

**Fig. 5:**
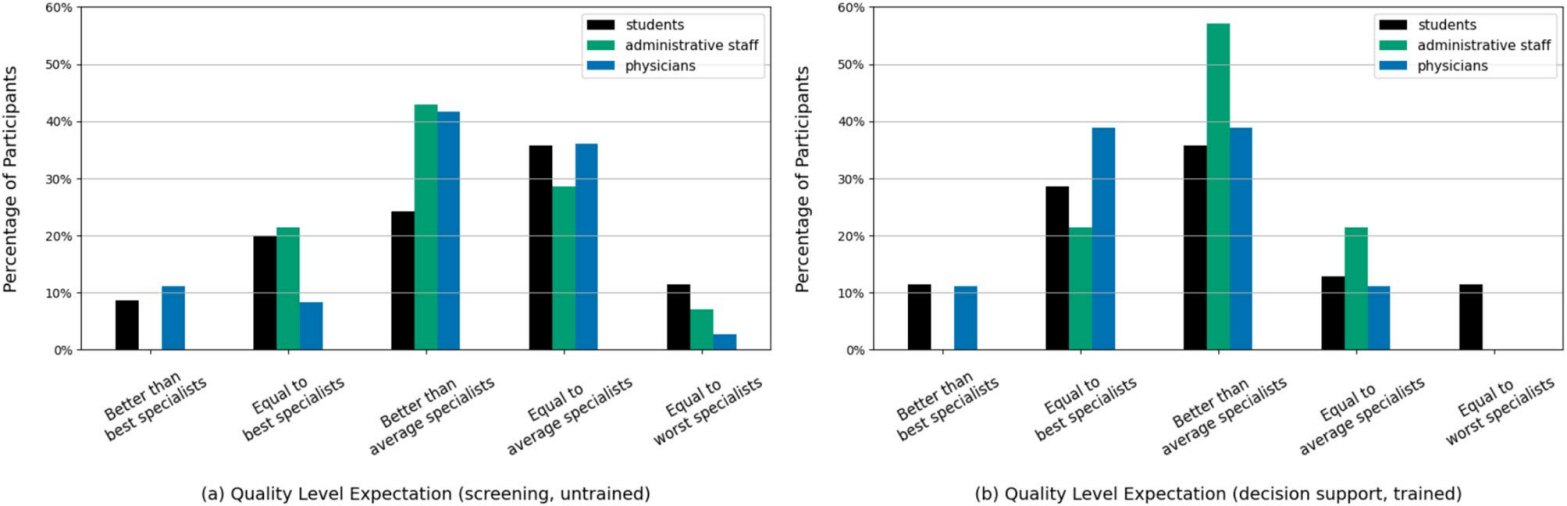
Left plot (**a**):“If a language model were to be used for screening purposes by untrained healthcare personnel in your field of expertise, what level of error in the model’s assessments do you consider acceptable?”Right plot (**b**):“What level of error in the assessments of a language model would be acceptable if it were used by trained professionals/physicians as diagnostic decision support?”.

We questioned the participants about the required performance standards for LLMs (Fig. 5) in two different scenarios with two different medical groups. In the left plot, the participants were asked about the performance standards necessary when untrained healthcare personnel (like medical technicians) use the LLMs for general screening purposes like disease detection. Here, a considerable proportion of all respondents (n=52; 43.33%) find the LLM screening performance equal to the level of or even below the level of an average specialist to be sufficient.

On the other hand, in the right plot, participants were asked about the required performance of LLMs when used by trained professionals (like physicians) for diagnostic decision support. In this case, only (n=24; 20.0%) deemed an average or below-average performance of the LLM systems to be sufficient. Instead, the vast majority (n=96; 80.0%) responded that LLM systems should exceed the proficiency of at least an average specialist.

Approval of Example LLM Use Scenario In another survey question, the following workflow procedure was suggested: “*During a pandemic, a specialist responds to patient questions online. To save time, they generate responses with a language model and then only review them before sending*”. The large majority of the participants (n=95; 79.17%) were open to adopting this method, with physicians being the group most in favor of the approach (n=33; 91.67%). No physicians answered“no”to this question. This question was tailored to the focus of the biomedical NLP research community on question-answering tasks.

### Opportunities and risks

**Fig. 6:**
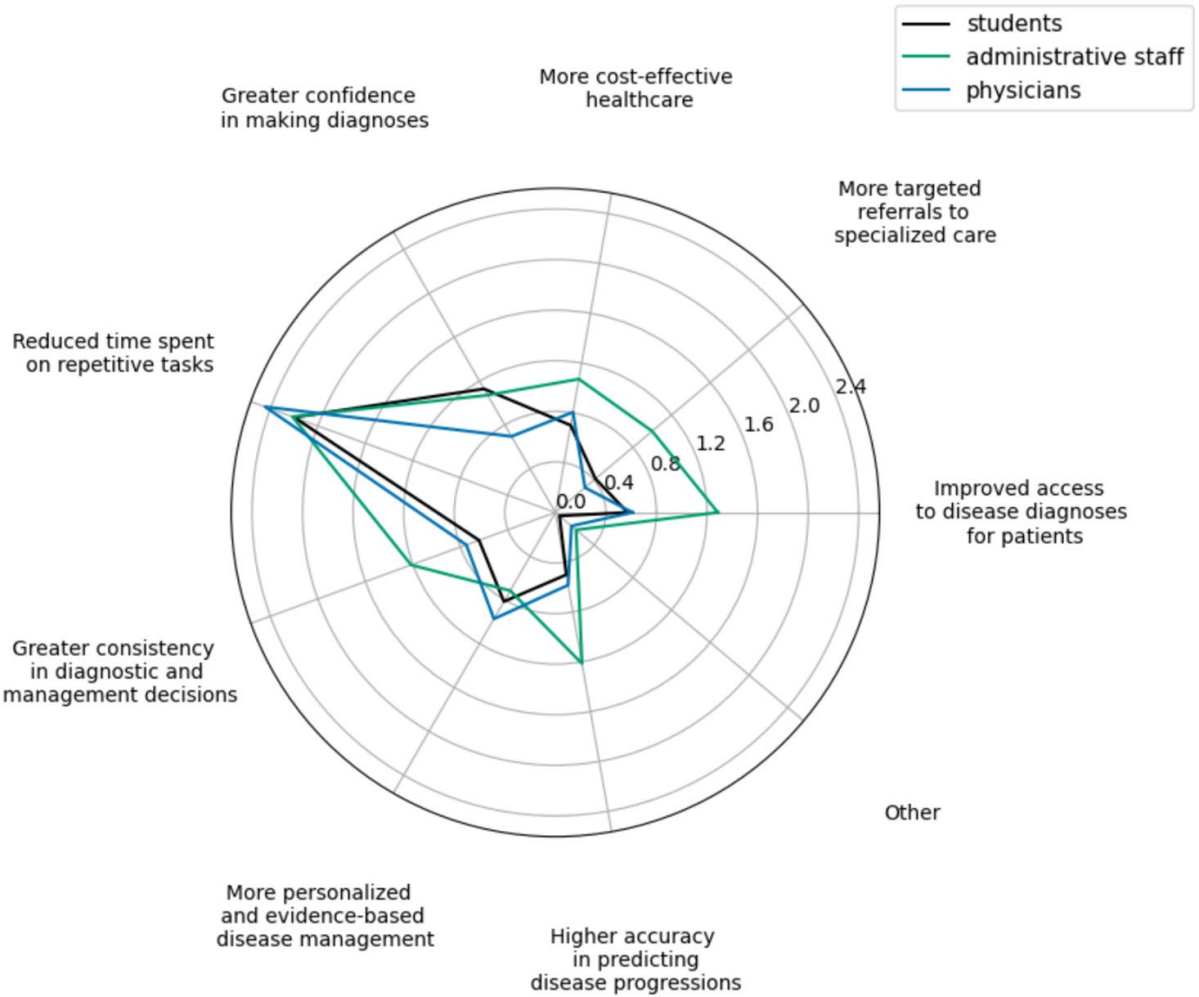
“Which of the following do you perceive as the greatest potential advantage of the use of language model systems in your field?”Participants indicated their top three preferences from a list of set choices. Higher scores in the radar plot indicate a higher ranking.

**Fig. 7:**
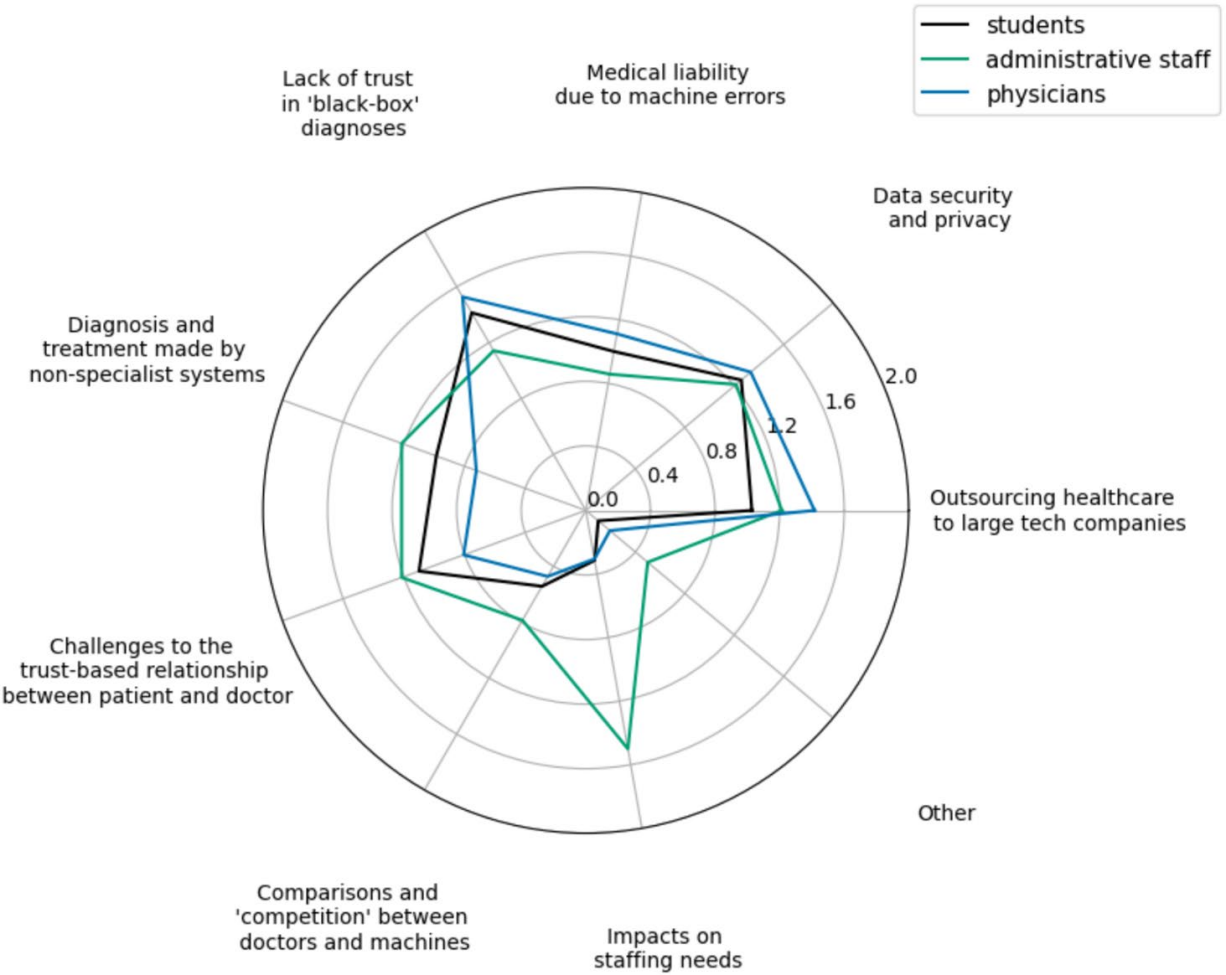
“Which of the following do you perceive as concerns to the utilization of language models in your field?”Participants indicated their top three concerns from a list of set choices. Participants indicated their top three preferences from a list of set choices. Higher scores in the radar plot indicate a higher ranking.

Two large-scale questions asked the participants to rank potential advantages and concerns of using LLMs in their field. Figures 6 and 7) show radar plots that visualize the responses.

#### Perceived advantages and benefits

As visualized in Fig. 6, all three occupational groups agreed that the most important benefit of introducing LLMs in the medical field lies in the potential to reduce time spent on monotonous and repetitive tasks (n=78, 65% responses ranked as 1st). The next two benefits that physicians rated as the most influential are“more personalized and evidence-based disease management”and“more cost-efficient healthcare”. On the other hand, students found the second most important benefit to be“greater confidence in making diagnoses”, while the third place was a close call between“more cost-efficient healthcare”and“greater consistency in diagnosis and management decisions”. Finally, administrative staff rated very closely“improved access to disease diagnoses for patients”and“higher accuracy in predicting disease progressions”.

#### Perceived risks and concerns

 When it comes to the perceived risks and concerns (Fig. 7), physicians found the most important ones to be: (1) lack of trust in “black-box”(nontransparent) diagnoses, (2) outsourcing healthcare to large tech companies, and (3) data security and privacy concerns. Students also found the (1) lack of trust in“black-box”diagnoses to be the most important, followed by (2) data security and privacy, and (3) challenges to the trust-based relationship between patients and doctors. Finally, administrative staff found the biggest concern to be the impact on staffing needs, which was rated in last place by physicians and students. Tied from second to fourth place for the administrative staff were diagnosis and treatment made by non-specialist systems, outsourcing healthcare to large tech companies, and data security and privacy concerns.

### Preparedness of medical centers and university clinics for introduction of LLMs

Most participants of our survey (n=77; 64.17%) believe that their institution (university/clinic/medical center) is not adequately equipped to deal with the introduction of LLMs. A much lower number of respondents (n=14; 11.67%) think their institutions are adequately prepared.

In a free-form followup question, we asked the participants on suggestions on what has to change for better preparedness of their institutions for the introduction of LLM technologies. Even though the question was optional, we received a large number of responses (n=78; 65%), showing the topic is important to participants and they would like to be involved in the process. We provide the most common themes and factors for adoption in Table 1. Illustrative quotes for most common themes are given in Supplementary Table 3.

**Table 1:** Overview of the most common key factors that survey respondents mentioned for improving the preparedness of their institutions for AI adoption.

**ID**	Key factor	Mentions
F1	Education about AI	46
F2	Ensure privacy & security	17
F3	Infrastructure & resources	13
F4	Provide examples, onboarding	10
F5	Digitalization of clinical texts	8
F6	Legal compliance	8
F7	Integration with hospital systems	6
F8	High accuracy of predictions	5
F9	Cooperation with IT departments	4
F10	Reduce bureaucracy	3
F11	Work with local language	3

As seen in the thematic content-analysis matrix in Table 1, the largest number of participants (38% of all and 59% of those who answered, n=46) identified education about AI and LLMs as the most crucial factor for successful adoption. This included responses such as the need for more seminars and courses about the abilities and potential of LLMs (one participant mentioned that“integrating the use of language models into the medical curriculum makes it easier to apply them later on”). The second most mentioned factor was Ensuring Privacy and Security of AI systems. The participants pointed out how medical paperwork often deals with highly confidential patient records and sensitive information like medical history or disease diagnoses. Another point was ensuring the security of LLM systems against any potential malicious use or adversarial attacks.

The third most popular factor was acquiring the necessary technical infrastructure and resources to host and support the deployed AI systems. The fourth most popular factor was the need for on-boarding and clear instructions of how and when to use the LLM-based tools in medical settings. Many respondents emphasized that they cannot be expected to start using the system out of the box without proper instructions and examples. Furthermore, another important factor was the digitalization of clinical text. Eight participants informed us that a significant portion of the text they use is still in handwritten format and has never been scanned or digitized (with one emphasizing“early organization of a data protection structure, digitization of clinical findings, digitization of dialysis data”).

Legal compliance, with eight mentions, emphasizes the need to respect and clarify legal regulations before introducing LLM systems into clinical practice. Integration within existing hospital systems (six mentions) refers to the need of seamlessly integrating newly adopted AI systems into those systems already in use. The requirement for high accuracy of predictions was mentioned five times, including considerations regarding liability for any medical errors.

Finally, four participants mentioned the need for good cooperation with existing IT departments of their respective institutions. Three responses focused on reducing the overall level of bureaucracy in the system as a prerequisite for wider AI adoption. Lastly, three respondents pointed out how, while LLMs work very well with the English language, they have to be properly tested and work well with the local language (German), considering that most clinical data will be provided in German.

## Discussion

Given the increasing amount of funding, research, and discussion dedicated to LLMs in multifold areas, it is essential to understand the expectations and needs of those using them. LLMs have the potential to transform healthcare and our study is among the first ones in the German context to investigate the opinions and attitudes of medical professionals toward the adoption of LLMs in medicine, especially with inclusion of medical students and administrative staff.

The introduction of LLMs is mostly seen as positively influencing their field. This follows the findings of related surveys on the use of AI in medicine^17,27,28^, but also newer ones on the use of LLMs by physicians and students^29–32^. Around one-half of both physicians and students in our study use LLMs daily or weekly. This finding highlights the growing recognition of the utility of LLMs in automating mundane tasks, thereby saving time and enabling higher-quality results. While they were the smallest participant group, administrative staff also had the lowest frequency of LLM use. The outreach and promotion of LLMs to help the medical community should also be more inclusive of administrative workers in the future. Finally, a considerable number of students said they have a worse understanding of the technology than their peers, suggesting that many feel left behind in the face of rapid digital developments.

Existing LLM use cases among respondents are diverse and multifaceted. Many utilize LLMs for general-purpose tasks, mirroring how the general population uses them^33^. Examples for such tasks are drafting emails, summarizing lengthy texts, translating articles, or conducting quick web searches. At the same time, the participants identified speech transcription and translating medical records into patients’ native languages as the most important use cases. Less frequently mentioned and perceived as less important were more complex medical tasks, like suggesting diagnoses or support in medical reasoning. This highlights the discrepancy in the focus of the NLP/AI community, which often prioritizes researching complex tasks over addressing the actual needs of medical workers. On the other hand, it is possible that medical experts are not informed of or acquainted with the possibilities that LLMs can provide and expect tools with user-friendly interfaces and instructions to start using them for more complex tasks. Finally, it is also evident that administrative staff rated as the most important those tasks that are closer to their daily work environment, such as speech transcription, document summarization, text and simplification, while rating the medical tasks like medical reasoning and concept extraction considerably lower than medical professionals.

The least popular LLM use case was using LLMs to answer patients’ questions. At first glance, this finding contradicts the popularity of the proposed workflow scenario of using LLMs to draft an answer to a patient question, where the vast majority agreed they would use LLMs for that purpose. This suggests that physicians are open to utilizing LLMs to assist in drafting well-structured responses to questions but highly prefer to verify and refine the content of the answers themselves.

The participants, especially physicians, mostly see the speed and level of impact of LLMs to be high. Interestingly enough, students were more moderate and careful in their assessment, with only a small group experiencing the current impact of LLMs. Since their medical careers are just about to commence and are expected to span multiple decades, they likely feel that there is enough time to adapt to the upcoming changes. Overall, students expect the workforce needs to change much less than physicians and administrative staff in both scenarios.

Another interesting observation was that most students and physicians did not expect a very high performance standard of LLMs for routine screening tasks (like disease detection from anamnesis) but overwhelmingly required a high standard when helping them with diagnostic decisions and treatment plans. This reflects the lower potential risk associated with routine tasks even if model predictions are not highly accurate. Intuitively, untrained professionals could be less likely to spot intricate medical errors in LLM outputs. Still, this was not seen as detrimental in the screening phase because any error can be corrected in later patient treatment phases. Therefore, LLM-based digital assistants could fill the role of trainees for routine tasks when helping physicians but be like a peer when used for the more critical task of decision support in patient treatment. Nevertheless, recent studies have shown that LLMs perform better in routine classification tasks while struggling in realistic patient diagnostics and adhering to clinical guidelines^12,34^, highlighting a mismatch between actual capabilities of current LLMs and expected error rate thresholds from survey participants.

All participant groups overwhelmingly see the reduced time spent on repetitive tasks as the biggest potential of LLMs in their work. This follows our insight on how they currently use LLMs and how they rated potential LLM use cases involving time-consuming tasks. Moreover, both physicians and students appreciated the opportunity of LLMs to lead to more cost-effective healthcare. By automating certain mundane tasks of everyday medical work, more time and energy of physicians can be directed to actual patient treatment, thus lowering the average cost of healthcare services^35^. Similarly, rural healthcare providers could utilize LLMs for support instead of spending expenses on getting help from metropolitan institutions^36^. Furthermore, physicians found important the idea of a more personalized and evidence-based disease management with LLMs, following the increasing trend in medicine on providing personalized treatment for each patient^37^. One way in which LLMs can help physicians here is, given a specific patient case, sifting through relevant medical sources like lengthy clinical guidelines or systematic reviews and summarizing the key points concisely to assist them in diagnosis and treatment.

The biggest concern that physicians and students have with LLMs is the low trust in“black-box”diagnoses, i.e., the lack of transparency about how LLMs work internally. This highlights the need to augment LLMs with methods that clarify their decision-making process, such as decision trees^38^, symbolic logic reasoning^39^, chain-of-thought mechanism^40^, and other techniques of *explainability*^41^. Recent advanced LLMs like OpenAI o3 or DeepSeek R1^42^ explain their reasoning by“thinking out loud”while generating an answer, making the generation process more transparent. Additionally, retrieval-augmented generation (RAG)^43^ can make the generated text grounded in trustworthy sources by linking facts in LLM answers to exact passages from medical guidelines^44^ or by fact-checking the answers^45^. Such measures are also important in the light of the so-called“control problem”^46^, which evidences humans becoming complacent with and over-reliant on reliable autonomous systems, leading to less rigorous manual checking of outputs.

Another significant concern for physicians was outsourcing healthcare to large technology companies, which have developed numerous popular large language models (LLMs), highlighting the need for investment in locally developed and tailored solutions for individual clinics. Furthermore, a concern highly ranked by all three groups was privacy considerations – as the medical domain often works with sensitive patient data, it is paramount to ensure their personal information will be protected^47^. On top of that, students highlighted the loss of the fiduciary relationship between patients and doctors, which could arise if machines are the ones making the final decisions. Finally, administrative workers had the impact on workforce needs as their highest concern. One reason for this spike could be that they are particularly afraid of their work being automated, but this has to be confirmed in further research before conclusions can be drawn. Nevertheless, the role of LLMs as tools that assist humans instead of fully automating work should be emphasized when adopting the technology and in educational training.

One major finding of our survey was the negative feeling about the level of preparedness of participants’ institutions for the introduction of LLMs and AI into work. More than 80% of physicians feel their clinics are not ready for it, with only around 10% of both students and physicians thinking they are ready. When asked how to improve it, the majority of all respondents found it crucial to provide proper education and onboarding for employees on how LLMs work and what they can achieve and be used for. We encourage future work and efforts to increase public outreach and organize seminars and tutorials where medical personnel would be introduced to the potential and practical use of LLMs. Other crucial topics that training should cover are best practices and caveats of prompting, as well as the aforementioned control problem.

Another common factor for successful adoption was ensuring a high level of privacy and security, as well as compliance of models with laws and regulations. This can be achieved by deploying local LLMs, which would be run on private servers belonging to clinics and universities, thus ensuring that no sensitive data leaves the premises and providing autonomy over the AI tools. Similar recurring themes included investment in proper technical infrastructure, digitalization of paper-written clinical texts, proper coordination with local IT departments, and integration within existing hospital systems that workers are already accustomed to. However, especially the last fact could prove to be a significant challenge in Germany, as there is a lack of standardization in IT systems across clinics^48^. Finally, a smaller number of responses highlighted the need for high output factuality and also good LLM capabilities in the native language of those using the models.

The finding of the institutional lack of preparedness to introduce LLMs stands in contrast to the existing frequent usage of LLMs by physicians. Based on statements provided in answers, this discrepancy is mostly explained by the private use of LLMs for work when the clinic does not provide any guidelines. At the same time, this represents a great risk to privacy and data security (since there is no regulation or training) and shows the high value of LLMs to physicians who are ready to accept this risk. A similar situation is present in the adoption of legal AI in law firms, where lawyers already use LLMs personally to help them with email drafting and legal cases, but firms often do not regulate or mandate how to use it^49^.

Considering that many questions in our survey were based on the study by Scheetz and Rothschild et al.^50^, conducted in 2019 and focusing on AI for medical imaging, we can draw some comparisons. While only 20% of physicians in their survey actively used AI tools, 50% did so in ours – this shows the general rise in popularity of AI driven by user-friendly chat interfaces and widespread promotion of LLMs. While participants in both surveys agreed the impact of AI on their field is positive, more respondents agreed in their survey (71%) compared to ours (57%). This could point to the fact that the higher popularity of LLMs comes with a higher awareness of potential risks and liabilities associated with them. One notable difference is the question on preparedness of the hospitals for AI adoption (34% said“not prepared”in theirs and 83% in ours), showing the different degree of digitalization and challenges with AI adoption between the two regions. Another major difference was their finding of“improved patient access to disease screening”as the most promising benefit selected by physicians, which was not among top 3 in our survey. This is likely because visual models are mostly used in medicine to detect disease from imaging, while LLMs have a wider range of applications. Their survey also showed a two times higher percentage of respondents concerned about the change in workforce needs – the lower number in our survey might indicate that better familiarity with LLMs also comes with recognizing their limitations and how they mainly assist but cannot fully automate clinical work.

Looking at the path forward, there are several important actionable considerations. There is a large need for organizing educations, seminars, and practical courses on the use of AI targeted towards the medical community and adapted for different groups and use cases. Three medical groups we investigated showed different needs and concerns, therefore the developed solutions should be tailored to different groups or provide a certain filter (mask) to select only those features relevant to that group. Furthermore, the developed systems should include a degree of explainability of their decisions, which can be achieved by grounding responses in trustworthy sources, automated fact-checking, reasoning chains, and other symbolic approaches that enhance interpretability. The current hospital systems should be digitalized, including documentation and guidelines, for easier integration of more sophisticated AI tools into their systems. Any developed AI solutions have to respect the privacy of patient data, which can be achieved through anonymization techniques and methods like federated learning. Finally, physicians, students, and staff should always be included and consulted in the solution development process.

We acknowledge several limitations of our study. Filling out the survey was completely voluntary and anonymous, so the sample is not representative. The relatively low participant count for the administrative staff group adds to this issue, so the statistical results should be interpreted carefully. Subgroup differences or reported percentages may reflect random variation rather than true patterns in the broader population. Moreover, the sample is likely biased toward technology-savvy participants who already use LLMs and were more inclined to complete the survey. Additionally, the high proportion of medical students and the choice of university clinics (as opposed to non-academic and rural hospitals) likely skews the sample toward a younger and more digitally engaged demographic. This may limit generalizability and the lack of readiness for AI adoption that we identified is likely even more severe in the broader, non-academic workforce. Despite this tech-savvy bias, 30% of respondents still indicated they never use LLMs. Another limitation is the study’s limited geographic scope, as all participants are based in Germany, specifically in Bavaria. German health-system governance is decentralized, involving both the German federal and state governments, which can limit generalizability to the rest of Germany. Therefore, similar studies in other regions could help in evaluating the generalizability of our findings. A similar concern is the timeliness of results, since the survey was conducted in 2024 and AI is a technically fast-moving field so people’s perceptions of the technology can shift rapidly. An additional threat to validity is the potential for researcher bias, as some of the coding procedures and discussion points in our study involve subjective interpretations. We aimed to mitigate this by discussing the findings with a team of three researchers, limiting individual biases. Finally, the findings may be affected by social desirability bias, meaning the data and derived findings are based on the information the survey respondents wanted to share with us and may not necessarily capture the complete picture.

## Conclusion

In this study, we conducted a questionnaire survey with the aim of investigating the expectations and opinions of the medical community in Germany on the use of increasingly popular large language models in their daily work. In total, 70 medical students, 36 physicians, and 14 administrative workers shared their thoughts about potential use cases, perceived degree of impact, quality expectations, advantages and risks, and factors for future adoption of LLMs in medicine. Many participants already use LLMs as a tool for research support, as well as summarization, translation, or report drafting, and see patient-centered use cases like simplification and translation of medical reports as particularly relevant. Most participants see LLMs as having a positive influence on their field and assess that the impact is already present and moderately high. They greatly appreciate using LLMs to save time by automating repetitive tasks involving written text, which can lead to more cost-effective and personalized patient treatment. Concerns were shown regarding the opaque nature of LLMs’ inner workings, confidential data privacy, the influence of large tech companies, and the potential loss of trust between patients and physicians. Participants overwhelmingly do not find their current institution well-equipped for the introduction of LLMs. Suggestions to bridge this gap include offering broader education and specialized training on the use of LLMs in medicine, making higher investments in digitalization, ensuring good infrastructure, standardizing IT systems, ensuring legal compliance and data protection, and fostering a culture of technological openness. We hope that our work will encourage better-informed design and development of future LLM-based tools for medical workers, taking into account their needs, thoughts, and requirements, leading to a better quality of patient treatment and higher job satisfaction of medical personnel.

## Methods

### Survey design

Our study was based on an anonymous online survey in the form of a questionnaire. The survey consisted of 22 questions, out of which 17 were multiple-choice questions, and 5 were questions with free-form input. We separated questions into four logical blocks. The first block asks basic occupational questions and inquires about whether the participant already uses LLMs – if yes, for which use cases – and how relevant were some selected use cases in their daily work. The second question block revolves around the opinion on how soon the LLMs will have a noticeable impact in their field and how it will affect the workforce. The third question block asks about how ready they think their institution is to adopt AI technologies and what kind of error level and quality the language models should have to be helpful. The fourth question block focuses on the perceived benefits and perceived risks of the adoption of LLMs in their field. The final question is for any additional remarks on the survey. The survey questions were originally written in German, however, for this paper, we translated all questions and answers to English. All the original German and translated English questions can also be found in Supplementary materials. The majority of questions were based on the existing survey from Scheetz and Rothschild et al.^50^, conducted in 2019 on the use of vision AI among physicians. This allowed, among other findings, to compare how the attitudes have changed in the last five years and how different opinions on LLMs are compared to computer vision tools. After adapting the existing questions to be specific to LLM usage, we performed a pilot run and checks with two in-house university physicians and modified or rewrote any questions that were unclear.

### Data collection

The questionnaire was primarily hosted on Bayern Collab^51^, a safe and secure server based on Confluence software and managed by Digitalverbund Bayern (https://digitalverbund.bayern). Confluence is a web-based corporate wiki software, and Digitalverbund Bayern is the official association of Bavarian universities and higher education institutions. The platform Bayern Collab was recommended by the Ethical Committee of the Technical University of Munich.

The users had to use their university credentials to log in to the platform, but the data collection process itself was completely anonymous. The Bayern Collab platform records only the timestamp of a survey response, which is completely unidentifiable. The survey was open and accepting answers for six months, from April 2024 to October 2024. The collected electronic data was stored on a password-protected and backed-up computer drive on the safe server and remained confidential – only the two principal investigators (paper authors) had access to it and used it to run statistical descriptive analyses on the aggregated answers, to understand and interpret the results.

### Survey distribution

The survey was targeted at three professional groups: medical doctors, medical administrative personnel, and students of medicine. The survey was distributed at five Bavarian university clinics: TUM University Clinic – Klinikum Rechts der Isar (MRI), LMU University Clinic (LMU Klinikum), University Clinic Augsburg, University Clinic Regensburg, and University Clinic Würzburg. The survey was distributed through official channels and intranet postings at the respective clinics and targeted at the three surveyed groups. Owing to the anonymous nature of the survey, we cannot distinguish how many responses came from which of the clinics. Our assumption is that the majority comes from the TU Munich, as the starting point of the study, and the other clinics are somewhat equally represented.

### Ethical approval and consent

The study was performed in accordance with the Declaration of Helsinki. The prospective online survey was officially approved by the Ethical Committee of the Technical University of Munich^52^ (decision number: 2023-596-S) and by the Data Protection Office of the Technical University of Munich^53^ (decision number: VT-1057). These approvals were valid for the conduction of the survey on other Bavarian university clinics as well. Before starting the survey, each participant gave electronic informed consent and was also provided with a 4-page document describing the consent and how the data will be stored and analyzed (generated by the official tool *eTIC –- electronic Tool for the compilation of Informed Consent documents*^54^). Additionally, participants were provided with a two-page study protocol describing the purpose and conduction of the study, as well as a one-page document outlining a brief introduction to the most recent advancements in biomedical NLP and a description of how LLMs work. These are provided as attachments to our submission.

### Data analysis methods

Only fully completed survey responses were taken into consideration for data analysis. The data was analyzed using Python libraries *numpy*, *pandas*, and *scipy*, while the plots were generated with the Python library *matplotlib*. Categorical responses were compared according to the participants group using Pearson’s $\chi ^2$ test, for the test of independence of the observed frequencies in contingency tables, while its effect size was estimated with Cramér’s *V*. Ordinal responses (Likert scale 1–5) were compared with regard to participant groups using the Kruskal-Wallis test to test whether samples originate from the same distribution, while its effect size was estimated with $\eta ^2$. To determine the most common themes in questions related to the existing use cases of LLMs and suggestions for successful LLM adoption, we employed the hybrid thematic analysis approach. Two authors followed a six-step framework proposed by Braun and Clarke^55^: (1) explore the free-form answers; (2) generate initial codes for observed phenomena; (3) identify potential themes and sub-themes in codes; (4) review and rephrase; (5) identify the overarching themes and merge similar ones; (6) write up a narrative. The codes were generated with an inductive, data-driven approach independently by the two authors after reading and reviewing the responses. Any discrepancy in coding was resolved through discussion and consensus in regular weekly meetings, where codes were discussed, clarified, and refined. To supplement our qualitative interpretation, the final themes were quantified through content analysis to determine their frequency in the dataset. To evaluate the responses to questions related to the benefits and risks of LLMs, we used a scoring system where the 1st ranked choice was given 3 points, 2nd choice 2 points, and 3rd choice 1 point. The total score was averaged by the number of participants in a group.

## Supplementary Information

Supplementary Information 1.

Supplementary Information 2.

## Data Availability

All data generated or analyzed during this study, including the raw export of all survey responses, are included in this published article and its supplementary information files.
