# Supplementary material for: Investigating expectations and needs regarding the use of large language models at Bavarian university clinics
Source: Sci Rep. 2026 Mar 26;16:10505. doi: 10.1038/s41598-026-45245-2 (PMC13031305; doi:10.1038/s41598-026-45245-2)
Supplement: Supplementary file 1 — Supplementary Information 1. [file 41598_2026_45245_MOESM1_ESM.pdf]

**Supplementary Tables**

**Title:** Investigating Expectations and Needs of Medical Professionals Regarding the Use of Large Language Models: A Study at German University Clinics

**Venue:** Scientific Reports

**Authors**: Juraj Vladika*, Alexander Fichtl, Florian Matthes

*Corresponding Author (juraj.vladika@tum.de)

| Supplementary Table 1: Survey questions in German (original) and English (translated) with corresponding answer frequency per participant group | | | | | | |
|---|---|---|---|---|---|---|
| **Question (German)** | **Question (English)** | **Answer (German)** | **Answer (English)** | **Physicians** | **Students of Medicine** | **Administrative Staff** |
| ***Number of participants (n)*** | | | | 36 | 70 | 14 |
| **Was ist Ihr medizinisches Fachgebiet? (falls zutreffend)** | **What is your medical specialty? (if applicable)** | **Neurologie** | **Neurology** | 3 (8.3%) | 1 (1.4%) | 0 (0%) |
| | | **NaN** | **NaN** | 4 (11.1%) | 65 (92.9%) | 9 (64.3%) |
| | | **Algemein- und Viszeralchirurgie** | **General and visceral surgery** | 1 (2.8%) | 0 (0%) | 0 (0%) |
| | | **Urologie** | **Urology** | 2 (5.6%) | 0 (0%) | 0 (0%) |
| | | **Tierpathologie** | **Animal pathology** | 1 (2.8%) | 0 (0%) | 0 (0%) |
| | | **Psychosomatik** | **Psychosomatics** | 1 (2.8%) | 0 (0%) | 0 (0%) |
| | | **Innere Medizin** | **Internal medicine** | 2 (5.6%) | 0 (0%) | 0 (0%) |
| | | **Psychiatrie und Psychotherapie** | **Psychiatry and psychotherapy** | 4 (11.1%) | 0 (0%) | 0 (0%) |
| | | **Psychiatrie** | **Psychiatrie** | 4 (11.1%) | 0 (0%) | 0 (0%) |
| | | **Innere Mediyin** | **Inner Mediyin** | 1 (2.8%) | 0 (0%) | 0 (0%) |
| | | **Nephrologie** | **Nephrology** | 2 (5.6%) | 0 (0%) | 0 (0%) |
| | | **Radiologie** | **Radiology** | 1 (2.8%) | 0 (0%) | 0 (0%) |
| | | **Medizinethik** | **Medical ethics** | 0 (0%) | 1 (1.4%) | 0 (0%) |
| | | **Arbeitsmedizin** | **Occupational medicine** | 0 (0%) | 0 (0%) | 1 (7.1%) |
| | | **Entwickler** | **Developer** | 0 (0%) | 0 (0%) | 1 (7.1%) |
| | | **HNO** | **ENT** | 2 (5.6%) | 0 (0%) | 0 (0%) |
| | | **Psychiatrie, Somatik** | **Psychiatry, somatics** | 0 (0%) | 0 (0%) | 1 (7.1%) |
| | | **Psychiatrie und Somtomatik** | **Psychiatry and somtomatics** | 0 (0%) | 0 (0%) | 1 (7.1%) |
| | | **Neurologie / Psychiatrie** | **Neurology / Psychiatry** | 1 (2.8%) | 0 (0%) | 0 (0%) |
| | | **Weiterbildung Psychiatrie** | **Further training in psychiatry** | 1 (2.8%) | 0 (0%) | 0 (0%) |
| | | **Neuroradiologie** | **Neuroradiology** | 1 (2.8%) | 0 (0%) | 0 (0%) |
| | | **Studiengangsentwicklung** | **Course development** | 0 (0%) | 0 (0%) | 1 (7.1%) |
| | | **Allgemeinmed** | **General medicine** | 0 (0%) | 1 (1.4%) | 0 (0%) |
| | | **Nuklearmedizin** | **Nuclear medicine** | 1 (2.8%) | 0 (0%) | 0 (0%) |

| | | | | | | |
|---|---|---|---|---|---|---|
| | | **Plastische, Hand- und wiederherstellungschirurgie** | **Plastic, hand and reconstructive surgery** | 1 (2.8%) | 0 (0%) | 0 (0%) |
| | | **chirurgie/orthopädie** | **surgery/orthopaedics** | 1 (2.8%) | 0 (0%) | 0 (0%) |
| | | **Masterstudentin Translational Medicine** | **Masterstudentin Translational Medicine** | 0 (0%) | 1 (1.4%) | 0 (0%) |
| | | **Orthopädie und Unfallchirurgie** | **Orthopaedics and trauma surgery** | 1 (2.8%) | 0 (0%) | 0 (0%) |
| | | **hno** | **ent** | 1 (2.8%) | 0 (0%) | 0 (0%) |
| | | **Psychiatrie, Neurologie** | **Psychiatry, neurology** | 0 (0%) | 1 (1.4%) | 0 (0%) |
| **Berufserfahrung in Ihrem Fachgebiet in Jahren?** | **Years of professional experience in your field?** | **NaN** | **NaN** | 9 (25%) | 48 (68.6%) | 3 (21.4%) |
| | | **5-10 Jahre** | **5-10 years** | 9 (25%) | 5 (7.1%) | 4 (28.6%) |
| | | **<5 Jahre** | **<5 years** | 5 (13.9%) | 15 (21.4%) | 0 (0%) |
| | | **10-20 Jahre** | **10-20 years** | 9 (25%) | 2 (2.9%) | 2 (14.3%) |
| | | **20-30 Jahre** | **20-30 years** | 3 (8.3%) | 0 (0%) | 4 (28.6%) |
| | | **>30 Jahre** | **>30 years** | 1 (2.8%) | 0 (0%) | 1 (7.1%) |
| **Wie oft nutzen Sie auf Sprachmodellen basierende Software (wie z.B. ChatGPT) in Ihrem Fachgebiet?** | **How often do you use software based on language models (such as ChatGPT) in your field?** | **Monatlich** | **Monthly** | 6 (16.7%) | 16 (22.9%) | 1 (7.1%) |
| | | **Wöchentlich** | **Weekly** | 12 (33.3%) | 23 (32.9%) | 1 (7.1%) |
| | | **Nie** | **Nie** | 10 (27.8%) | 18 (25.7%) | 6 (42.9%) |
| | | **Täglich** | **Daily** | 6 (16.7%) | 10 (14.3%) | 4 (28.6%) |
| | | **Ich bin mir nicht sicher** | **I am not sure** | 2 (5.6%) | 3 (4.3%) | 2 (14.3%) |
| **Wofür nutzen Sie (ggf) Sprachmodelle?** | **What do you use language models for (if anything)?** | *(free-text answers)* | | | | |
| **Wie relevant sind diese Anwendungsfälle von Sprachmodellen für Ihre tägliche Arbeit? (nicht nur für Ihre derzeitige Nutzung, sondern auch für die mögliche Nutzung in der Zukunft) [Extraktion von wichtigen Informationen und Beweisen aus Dokumenten wie medizinischen Forschungsunterlagen und Patientennotizen]** | **How relevant are these use cases of language models for your daily work? (not only for your current use, but also for potential use in the future) [Extraction of important information and evidence from documents such as medical research papers and patient notes]** | **1 - überhaupt nicht relevant** | **1 - not relevant at all** | 8 (22.2%) | 9 (12.9%) | 5 (35.7%) |
| | | **2 - eher nicht relevant** | **2 - rather not relevant** | 3 (8.3%) | 14 (20%) | 4 (28.6%) |
| | | **3 - neutral** | **3 - neutral** | 4 (11.1%) | 12 (17.1%) | 1 (7.1%) |
| | | **4 - eher relevant** | **4 - rather relevant** | 13 (36.1%) | 19 (27.1%) | 2 (14.3%) |
| | | **5 - sehr relevant** | **5 - very relevant** | 8 (22.2%) | 16 (22.9%) | 2 (14.3%) |
| **Wie relevant sind diese Anwendungsfälle von Sprachmodellen für Ihre tägliche Arbeit? (nicht nur für Ihre derzeitige Nutzung, sondern auch für die mögliche Nutzung in der Zukunft) [Beantwortung von Fragen** | **How relevant are these use cases of language models for your daily work? (not only for your current use, but also for possible use in the future) [answering patients' questions or helping doctors make** | **1 - überhaupt nicht relevant** | **1 - not relevant at all** | 11 (30.6%) | 18 (25.7%) | 7 (50%) |
| | | **2 - eher nicht relevant** | **2 - rather not relevant** | 11 (30.6%) | 23 (32.9%) | 1 (7.1%) |
| | | **3 - neutral** | **3 - neutral** | 2 (5.6%) | 10 (14.3%) | 2 (14.3%) |
| | | **4 - eher relevant** | **4 - rather relevant** | 7 (19.4%) | 17 (24.3%) | 2 (14.3%) |

| | | | | | | |
|---|---|---|---|---|---|---|
| **der Patienten oder Entscheidungshilfe für Ärzte in gesprächiger Form]** | **decisions in a conversational way].** | **5 - sehr relevant** | **5 - very relevant** | 5 (13.9%) | 2 (2.9%) | 2 (14.3%) |
| **Wie relevant sind diese Anwendungsfälle von Sprachmodellen für Ihre tägliche Arbeit? (nicht nur für Ihre derzeitige Nutzung, sondern auch für die mögliche Nutzung in der Zukunft) [Verbesserung der medizinischen Argumentation, z. B. Entdeckung von Krankheiten anhand von Symptomen]** | **How relevant are these use cases of language models for your daily work? (not only for your current use, but also for possible use in the future) [improvement of medical reasoning, e.g. detection of diseases based on symptoms].** | **1 - überhaupt nicht relevant** | **1 - not relevant at all** | 7 (19.4%) | 10 (14.3%) | 6 (42.9%) |
| | | **2 - eher nicht relevant** | **2 - rather not relevant** | 6 (16.7%) | 7 (10%) | 3 (21.4%) |
| | | **3 - neutral** | **3 - neutral** | 3 (8.3%) | 16 (22.9%) | 1 (7.1%) |
| | | **4 - eher relevant** | **4 - rather relevant** | 12 (33.3%) | 30 (42.9%) | 1 (7.1%) |
| | | **5 - sehr relevant** | **5 - very relevant** | 8 (22.2%) | 7 (10%) | 3 (21.4%) |
| **Wie relevant sind diese Anwendungsfälle von Sprachmodellen für Ihre tägliche Arbeit? (nicht nur für Ihre derzeitige Nutzung, sondern auch für die mögliche Nutzung in der Zukunft) [Transkription von Sprache in Textform]** | **How relevant are these use cases of language models for your daily work? (not only for your current use, but also for possible use in the future) [Transcription of speech to text]** | **NaN** | **NaN** | 0 (0%) | 1 (1.4%) | 0 (0%) |
| | | **1 - überhaupt nicht relevant** | **1 - not relevant at all** | 4 (11.1%) | 6 (8.6%) | 3 (21.4%) |
| | | **2 - eher nicht relevant** | **2 - rather not relevant** | 1 (2.8%) | 6 (8.6%) | 1 (7.1%) |
| | | **3 - neutral** | **3 - neutral** | 5 (13.9%) | 10 (14.3%) | 2 (14.3%) |
| | | **4 - eher relevant** | **4 - rather relevant** | 9 (25%) | 18 (25.7%) | 2 (14.3%) |
| | | **5 - sehr relevant** | **5 - very relevant** | 17 (47.2%) | 29 (41.4%) | 6 (42.9%) |
| **Wie relevant sind diese Anwendungsfälle von Sprachmodellen für Ihre tägliche Arbeit? (nicht nur für Ihre derzeitige Nutzung, sondern auch für die mögliche Nutzung in der Zukunft) [Übersetzung von medizinischen Berichten in die Muttersprache des Patienten]** | **How relevant are these use cases of language models for your daily work? (not only for your current use, but also for possible use in the future) [Translation of medical reports into the patient's native language]** | **1 - überhaupt nicht relevant** | **1 - not relevant at all** | 5 (13.9%) | 9 (12.9%) | 5 (35.7%) |
| | | **2 - eher nicht relevant** | **2 - rather not relevant** | 4 (11.1%) | 3 (4.3%) | 1 (7.1%) |
| | | **3 - neutral** | **3 - neutral** | 1 (2.8%) | 3 (4.3%) | 0 (0%) |
| | | **4 - eher relevant** | **4 - rather relevant** | 9 (25%) | 16 (22.9%) | 2 (14.3%) |
| | | **5 - sehr relevant** | **5 - very relevant** | 17 (47.2%) | 39 (55.7%) | 6 (42.9%) |
| **Wie relevant sind diese Anwendungsfälle von Sprachmodellen für Ihre tägliche Arbeit? (nicht nur für Ihre derzeitige Nutzung, sondern auch für die mögliche Nutzung in der Zukunft) [Vereinfachung der medizinischen Dokumente zum besseren Verständnis der Patienten]** | **How relevant are these use cases of language models for your daily work? (not only for your current use, but also for possible use in the future) [Simplification of medical documents for better patient understanding]** | **1 - überhaupt nicht relevant** | **1 - not relevant at all** | 6 (16.7%) | 11 (15.7%) | 5 (35.7%) |
| | | **2 - eher nicht relevant** | **2 - rather not relevant** | 2 (5.6%) | 5 (7.1%) | 1 (7.1%) |
| | | **3 - neutral** | **3 - neutral** | 5 (13.9%) | 11 (15.7%) | 0 (0%) |
| | | **4 - eher relevant** | **4 - rather relevant** | 15 (41.7%) | 28 (40%) | 2 (14.3%) |
| | | **5 - sehr relevant** | **5 - very relevant** | 8 (22.2%) | 15 (21.4%) | 6 (42.9%) |
| **Wie relevant sind diese Anwendungsfälle von Sprachmodellen für Ihre tägliche Arbeit? (nicht nur für Ihre derzeitige Nutzung, sondern auch für die mögliche Nutzung in der Zukunft) [Erstellung eines schriftlichen Berichts aus numerischen Daten (z. B. Blutkultur)]** | **How relevant are these use cases of language models for your daily work? (not only for your current use, but also for possible use in the future) [Creating a written report from numerical data (e.g. blood culture)]** | **1 - überhaupt nicht relevant** | **1 - not relevant at all** | 5 (13.9%) | 11 (15.7%) | 5 (35.7%) |
| | | **2 - eher nicht relevant** | **2 - rather not relevant** | 6 (16.7%) | 4 (5.7%) | 2 (14.3%) |
| | | **3 - neutral** | **3 - neutral** | 4 (11.1%) | 14 (20%) | 0 (0%) |
| | | **4 - eher relevant** | **4 - rather relevant** | 9 (25%) | 29 (41.4%) | 2 (14.3%) |
| | | **5 - sehr relevant** | **5 - very relevant** | 12 (33.3%) | 12 (17.1%) | 5 (35.7%) |
| | | **1 - überhaupt nicht relevant** | **1 - not relevant at all** | 6 (16.7%) | 7 (10%) | 4 (28.6%) |

| | | | | | | |
|---|---|---|---|---|---|---|
| **Wie relevant sind diese Anwendungsfälle von Sprachmodellen für Ihre tägliche Arbeit? (nicht nur für Ihre derzeitige Nutzung, sondern auch für die mögliche Nutzung in der Zukunft) [Zusammenfassen langer schriftlicher Dokumente, z.B. Patientennotizen]** | **How relevant are these use cases of language models for your daily work? (not only for your current use, but also for possible use in the future) [Summarizing long written documents, e.g. patient notes]** | **2 - eher nicht relevant** | **2 - rather not relevant** | 2 (5.6%) | 6 (8.6%) | 1 (7.1%) |
| | | **3 - neutral** | **3 - neutral** | 7 (19.4%) | 9 (12.9%) | 1 (7.1%) |
| | | **4 - eher relevant** | **4 - rather relevant** | 10 (27.8%) | 27 (38.6%) | 3 (21.4%) |
| | | **5 - sehr relevant** | **5 - very relevant** | 11 (30.6%) | 21 (30%) | 5 (35.7%) |
| **Wie würden Sie im Vergleich zu Ihren Kolleg:innen in Ihrem Fachgebiet Ihr Wissen über Sprachmodelle und deren Anwendung in Ihrem Fachgebiet einschätzen? (1: Sehr schlecht, 5: Sehr gut)** | **Compared to your colleagues in your field, how would you rate your knowledge of language models and their application in your field? (1: Very poor, 5: Very good)** | **1.0** | **1.0** | 6 (16.7%) | 5 (7.1%) | 2 (14.3%) |
| | | **2.0** | **2.0** | 8 (22.2%) | 17 (24.3%) | 4 (28.6%) |
| | | **3.0** | **3.0** | 3 (8.3%) | 27 (38.6%) | 4 (28.6%) |
| | | **4.0** | **4.0** | 13 (36.1%) | 18 (25.7%) | 2 (14.3%) |
| | | **5.0** | **5.0** | 6 (16.7%) | 3 (4.3%) | 2 (14.3%) |
| **Wie lange wird es Ihrer Meinung nach dauern, bis Sprachmodelle einen spürbaren Einfluss auf Ihr Fachgebiet haben werden?** | **How long do you think it will be before language models have a noticeable impact on your field?** | **NaN** | **NaN** | 13 (36.1%) | 34 (48.6%) | 5 (35.7%) |
| | | **5-10 Jahre** | **5-10 years** | 5 (13.9%) | 15 (21.4%) | 3 (21.4%) |
| | | **<5 Jahre** | **<5 years** | 8 (22.2%) | 9 (12.9%) | 0 (0%) |
| | | **Es gibt bereits einen spürbaren Einfluss** | **There is already a noticeable influence** | 9 (25%) | 12 (17.1%) | 5 (35.7%) |
| | | **>10 Jahre** | **>10 years** | 1 (2.8%) | 0 (0%) | 1 (7.1%) |
| **Wie stark werden sich Sprachmodelle im nächsten Jahrzehnt auf den Bedarf an Arbeitskräften in Ihrem Fachgebiet auswirken? (1: Keine Auswirkungen, 5: Starke Auswirkungen)** | **How much impact will language models have on the demand for workers in your field over the next decade? (1: No impact, 5: Strong impact)** | **1.0** | **1.0** | 5 (13.9%) | 10 (14.3%) | 0 (0%) |
| | | **2.0** | **2.0** | 7 (19.4%) | 23 (32.9%) | 2 (14.3%) |
| | | **3.0** | **3.0** | 6 (16.7%) | 21 (30%) | 5 (35.7%) |
| | | **4.0** | **4.0** | 9 (25%) | 10 (14.3%) | 3 (21.4%) |
| | | **5.0** | **5.0** | 9 (25%) | 6 (8.6%) | 4 (28.6%) |
| **Inwieweit werden Sprachmodelle über das nächste Jahrzehnt hinaus einen Einfluss auf den Bedarf an Arbeitskräften in Ihrem Fachgebiet haben? (1: Keine Auswirkungen, 5: Starke Auswirkungen)** | **To what extent will language models have an impact on the demand for workers in your field beyond the next decade? (1: No impact, 5: Strong impact)** | **1.0** | **1.0** | 4 (11.1%) | 5 (7.1%) | 0 (0%) |
| | | **2.0** | **2.0** | 2 (5.6%) | 20 (28.6%) | 2 (14.3%) |
| | | **3.0** | **3.0** | 12 (33.3%) | 23 (32.9%) | 5 (35.7%) |
| | | **4.0** | **4.0** | 9 (25%) | 13 (18.6%) | 2 (14.3%) |
| | | **5.0** | **5.0** | 9 (25%) | 9 (12.9%) | 5 (35.7%) |
| **Wie wird sich der Personalbedarf aufgrund von Sprachmodellen ändern?** | **How will staffing requirements change as a result of language models?** | **Verringerung** | **Reduction** | 15 (41.7%) | 28 (40%) | 9 (64.3%) |
| | | **Keine Änderung** | **No change** | 17 (47.2%) | 39 (55.7%) | 5 (35.7%) |
| | | **Erhöhung** | **Increase** | 4 (11.1%) | 3 (4.3%) | 0 (0%) |
| | | **Ich bin mir nicht sicher** | **I am not sure** | 3 (8.3%) | 24 (34.3%) | 2 (14.3%) |

| | | | | | | |
|---|---|---|---|---|---|---|
| **Glauben Sie, dass Ihre Universität/Klinik/Praxis ausreichend vorbereitet ist, um die Einführung von Sprachmodellen in Ihrem Fachgebiet zu bewältigen?** | **Do you believe that your university/clinic/practice is sufficiently prepared to handle the introduction of language models in your field?** | **Ja** | **Ja** | 3 (8.3%) | 7 (10%) | 4 (28.6%) |
| | | **Nein** | **No** | 30 (83.3%) | 39 (55.7%) | 8 (57.1%) |
| **Was sollte Ihrer Meinung nach Ihre Universität/Klinik/Praxis zur Vorbereitung auf den Einsatz von Sprachmodellen in Ihrem Fachgebiet tun?** | **What do you think your university/clinic/practice should do to prepare for the use of language models in your field?** | *(free-text answers)* | | | | |
| **Sprachmodelle könnten in Zukunft zur Erkennung von Krankheiten und zur Erteilung medizinischer Ratschläge verwendet werden. Wenn ein solches Sprachmodell von nicht ausgebildetem Gesundheitspersonal in Ihrem Fachgebiet verwendet werden sollte: Welcher Fehlergrad bei den Einschätzungen des Sprachmodells wäre Ihrer Meinung nach vertretbar?** | **Language models could be used in the future to identify diseases and give medical advice. If such a language model were to be used by untrained healthcare professionals in your field: In your opinion, what degree of error in the language model's assessments would be acceptable?** | **Besser als dem eines durchschnittlichen Spezialisten** | **Better than that of an average specialist** | 15 (41.7%) | 17 (24.3%) | 6 (42.9%) |
| | | **Gleich dem der besten Spezialisten** | **Equal to the best specialists** | 3 (8.3%) | 14 (20%) | 3 (21.4%) |
| | | **Besser als dem der besten Spezialisten** | **Better than the best specialists** | 4 (11.1%) | 6 (8.6%) | 0 (0%) |
| | | **Gleich dem eines durchschnittlichen Spezialisten** | **Equal to that of an average specialist** | 13 (36.1%) | 25 (35.7%) | 4 (28.6%) |
| | | **Gleich dem der schlechtesten Spezialisten** | **Equal to that of the worst specialists** | 1 (2.8%) | 8 (11.4%) | 1 (7.1%) |
| **Welcher Fehlergrad wäre bei den Einschätzungen eines Sprachmodells akzeptabel, wenn dieses von ausgebildetem Fachpersonal/Ärzt:innen als diagnostische Entscheidungsunterstützung eingesetzt wird?** | **What degree of error would be acceptable in the assessments of a language model when used by trained professionals/physicians as diagnostic decision support?** | **Besser als dem eines durchschnittlichen Spezialisten** | **Better than that of an average specialist** | 14 (38.9%) | 25 (35.7%) | 8 (57.1%) |
| | | **Gleich dem der besten Spezialisten** | **Equal to the best specialists** | 14 (38.9%) | 20 (28.6%) | 3 (21.4%) |
| | | **Besser als dem der besten Spezialisten** | **Better than the best specialists** | 4 (11.1%) | 8 (11.4%) | 0 (0%) |
| | | **Gleich dem eines durchschnittlichen Spezialisten** | **Equal to that of an average specialist** | 4 (11.1%) | 9 (12.9%) | 3 (21.4%) |
| | | **Gleich dem der schlechtesten Spezialisten** | **Equal to that of the worst specialists** | 0 (0%) | 8 (11.4%) | 0 (0%) |
| **Würden Sie den folgenden klinischen Arbeitsablauf in Betracht ziehen? Während einer Pandemie beantwortet ein Facharzt Fragen von Patienten online. Um Zeit zu sparen, generiert er Antworten mit einem Sprachmodell und überarbeitet diese dann nur noch vor dem Absenden.** | **Would you consider the following clinical workflow? During a pandemic, a specialist answers questions from patients online. To save time, he generates answers with a language model and then only revises them before sending them.** | **Ich bin mir nicht sicher** | **I am not sure** | 3 (8.3%) | 8 (11.4%) | 3 (21.4%) |
| | | **Ja** | **Ja** | 33 (91.7%) | 53 (75.7%) | 9 (64.3%) |
| | | **Nein** | **No** | 0 (0%) | 9 (12.9%) | 2 (14.3%) |
| **Welche drei der folgenden Punkte sehen Sie als den größten potenziellen Vorteil des Einsatzes von Sprachmodellen in Ihrem Fachgebiet? (geben Sie nur Ihre Top 3 an, wobei "1"** | **Which three of the following do you see as the greatest potential benefit of using language models in your specialty? (indicate only your top 3, where "1" = greatest** | **NaN** | **NaN** | 4 (11.1%) | 3 (4.3%) | 0 (0%) |
| | | **Nicht in Top 3** | **Not in top 3** | 20 (55.6%) | 46 (65.7%) | 6 (42.9%) |
| | | **1** | **1** | 3 (8.3%) | 6 (8.6%) | 4 (28.6%) |

| | | | | | | |
|---|---|---|---|---|---|---|
| = größter Vorteil) [Verbesserter Zugang zu Krankheitsdiagnosen für Patienten] | benefit) [Improved access to disease diagnoses for patients] | **2** | **2** | 4 (11.1%) | 7 (10%) | 2 (14.3%) |
| | | **3** | **3** | 5 (13.9%) | 8 (11.4%) | 2 (14.3%) |
| **Welche drei der folgenden Punkte sehen Sie als den größten potenziellen Vorteil des Einsatzes von Sprachmodellen in Ihrem Fachgebiet? (geben Sie nur Ihre Top 3 an, wobei "1" = größter Vorteil) [Gezieltere Überweisungen an fachärztliche Versorgung]** | **Which three of the following do you see as the greatest potential benefit of using language models in your specialty? (indicate only your top 3, where "1" = greatest benefit) [More targeted referrals to specialist care]** | **NaN** | **NaN** | 5 (13.9%) | 3 (4.3%) | 0 (0%) |
| | | **Nicht in Top 3** | **Not in top 3** | 26 (72.2%) | 53 (75.7%) | 8 (57.1%) |
| | | **1** | **1** | 2 (5.6%) | 3 (4.3%) | 4 (28.6%) |
| | | **2** | **2** | 2 (5.6%) | 9 (12.9%) | 0 (0%) |
| | | **3** | **3** | 1 (2.8%) | 2 (2.9%) | 2 (14.3%) |
| **Welche drei der folgenden Punkte sehen Sie als den größten potenziellen Vorteil des Einsatzes von Sprachmodellen in Ihrem Fachgebiet? (geben Sie nur Ihre Top 3 an, wobei "1" = größter Vorteil) [Kostengünstigere Gesundheitsversorgung]** | **Which three of the following do you see as the greatest potential benefit of using language models in your specialty? (indicate only your top 3, where "1" = greatest benefit) [More cost-effective healthcare]** | **NaN** | **NaN** | 3 (8.3%) | 4 (5.7%) | 0 (0%) |
| | | **Nicht in Top 3** | **Not in top 3** | 16 (44.4%) | 38 (54.3%) | 6 (42.9%) |
| | | **1** | **1** | 2 (5.6%) | 2 (2.9%) | 2 (14.3%) |
| | | **2** | **2** | 8 (22.2%) | 17 (24.3%) | 3 (21.4%) |
| | | **3** | **3** | 7 (19.4%) | 9 (12.9%) | 3 (21.4%) |
| **Welche drei der folgenden Punkte sehen Sie als den größten potenziellen Vorteil des Einsatzes von Sprachmodellen in Ihrem Fachgebiet? (geben Sie nur Ihre Top 3 an, wobei "1" = größter Vorteil) [Größere Sicherheit beim Stellen von Diagnosen]** | **Which three of the following do you see as the greatest potential benefit of using language models in your specialty? (indicate only your top 3, where "1" = greatest benefit) [Greater confidence in making diagnoses]** | **NaN** | **NaN** | 4 (11.1%) | 3 (4.3%) | 0 (0%) |
| | | **Nicht in Top 3** | **Not in top 3** | 19 (52.8%) | 31 (44.3%) | 7 (50%) |
| | | **1** | **1** | 3 (8.3%) | 17 (24.3%) | 3 (21.4%) |
| | | **2** | **2** | 6 (16.7%) | 9 (12.9%) | 2 (14.3%) |
| | | **3** | **3** | 4 (11.1%) | 10 (14.3%) | 2 (14.3%) |
| **Welche drei der folgenden Punkte sehen Sie als den größten potenziellen Vorteil des Einsatzes von Sprachmodellen in Ihrem Fachgebiet? (geben Sie nur Ihre Top 3 an, wobei "1" = größter Vorteil) [Reduzierter Zeitaufwand für monotone Aufgaben]** | **Which three of the following do you see as the greatest potential benefit of using language models in your field? (indicate only your top 3, where "1" = greatest benefit) [Reduced time spent on monotonous tasks]** | **NaN** | **NaN** | 0 (0%) | 1 (1.4%) | 0 (0%) |
| | | **Nicht in Top 3** | **Not in top 3** | 5 (13.9%) | 9 (12.9%) | 2 (14.3%) |
| | | **1** | **1** | 27 (75%) | 42 (60%) | 9 (64.3%) |
| | | **2** | **2** | 3 (8.3%) | 10 (14.3%) | 1 (7.1%) |
| | | **3** | **3** | 1 (2.8%) | 8 (11.4%) | 2 (14.3%) |
| **Welche drei der folgenden Punkte sehen Sie als den größten potenziellen Vorteil des Einsatzes von Sprachmodellen in Ihrem Fachgebiet? (geben Sie nur Ihre Top 3 an, wobei "1" = größter Vorteil) [Größere Einheitlichkeit bei Diagnose- und Managemententscheidungen]** | **Which three of the following do you see as the greatest potential benefit of using language models in your specialty? (indicate only your top 3, where "1" = greatest benefit) [Greater consistency in diagnostic and management decisions]** | **NaN** | **NaN** | 4 (11.1%) | 5 (7.1%) | 0 (0%) |
| | | **Nicht in Top 3** | **Not in top 3** | 17 (47.2%) | 42 (60%) | 6 (42.9%) |
| | | **1** | **1** | 3 (8.3%) | 6 (8.6%) | 4 (28.6%) |
| | | **2** | **2** | 6 (16.7%) | 10 (14.3%) | 1 (7.1%) |
| | | **3** | **3** | 6 (16.7%) | 7 (10%) | 3 (21.4%) |
| **Welche drei der folgenden Punkte sehen Sie als den größten potenziellen Vorteil des Einsatzes von** | **Which three of the following do you see as the greatest potential benefit of using language models** | **NaN** | **NaN** | 2 (5.6%) | 1 (1.4%) | 0 (0%) |
| | | **Nicht in Top 3** | **Not in top 3** | 16 (44.4%) | 35 (50%) | 9 (64.3%) |

| | | | | | | |
|---|---|---|---|---|---|---|
| **Sprachmodellen in Ihrem Fachgebiet? (geben Sie nur Ihre Top 3 an, wobei "1" = größter Vorteil) [Personalisierteres sowie evidenzbasierteres Krankheitsmanagement]** | **in your specialty? (indicate only your top 3, where "1" = greatest benefit) [More personalized and evidence-based disease management]** | **1** | **1** | 6 (16.7%) | 6 (8.6%) | 1 (7.1%) |
| | | **2** | **2** | 5 (13.9%) | 11 (15.7%) | 3 (21.4%) |
| | | **3** | **3** | 7 (19.4%) | 17 (24.3%) | 1 (7.1%) |
| **Welche drei der folgenden Punkte sehen Sie als den größten potenziellen Vorteil des Einsatzes von Sprachmodellen in Ihrem Fachgebiet? (geben Sie nur Ihre Top 3 an, wobei "1" = größter Vorteil) [Höhere Genauigkeit bei der Vorhersage von Krankheitsverläufen]** | **Which three of the following do you see as the greatest potential benefit of using language models in your field? (indicate only your top 3, where "1" = greatest benefit) [Greater accuracy in predicting disease progression]** | **NaN** | **NaN** | 4 (11.1%) | 1 (1.4%) | 0 (0%) |
| | | **Nicht in Top 3** | **Not in top 3** | 22 (61.1%) | 49 (70%) | 7 (50%) |
| | | **1** | **1** | 2 (5.6%) | 3 (4.3%) | 4 (28.6%) |
| | | **2** | **2** | 7 (19.4%) | 9 (12.9%) | 2 (14.3%) |
| | | **3** | **3** | 1 (2.8%) | 8 (11.4%) | 1 (7.1%) |
| **Welche drei der folgenden Punkte sehen Sie als den größten potenziellen Vorteil des Einsatzes von Sprachmodellen in Ihrem Fachgebiet? (geben Sie nur Ihre Top 3 an, wobei "1" = größter Vorteil) [Andere]** | **Which three of the following do you see as the greatest potential benefit of using language models in your field? (indicate only your top 3, where "1" = biggest advantage) [Other]** | **NaN** | **NaN** | 8 (22.2%) | 6 (8.6%) | 0 (0%) |
| | | **Nicht in Top 3** | **Not in top 3** | 24 (66.7%) | 62 (88.6%) | 12 (85.7%) |
| | | **2** | **2** | 2 (5.6%) | 1 (1.4%) | 1 (7.1%) |
| | | **3** | **3** | 2 (5.6%) | 1 (1.4%) | 1 (7.1%) |
| **Welche drei der folgenden Punkte sind Ihrer Meinung nach am problematischsten hinsichtlich der Verwendung von Sprachmodellen in ihrem Fachgebiet? (geben Sie nur Ihre Top 3 an, wobei "1" = größter Nachteil) [Bedenken hinsichtlich der Veräußerung des Gesundheitswesens an große Technologie- und Datenunternehmen]** | **Which three of the following do you think are most problematic regarding the use of language models in your specialty? (indicate only your top 3, where "1" = biggest drawback) [Concerns about selling healthcare to big tech and data companies]** | **NaN** | **NaN** | 2 (5.6%) | 3 (4.3%) | 0 (0%) |
| | | **Nicht in Top 3** | **Not in top 3** | 11 (30.6%) | 32 (45.7%) | 6 (42.9%) |
| | | **1** | **1** | 9 (25%) | 12 (17.1%) | 3 (21.4%) |
| | | **2** | **2** | 10 (27.8%) | 13 (18.6%) | 3 (21.4%) |
| | | **3** | **3** | 4 (11.1%) | 10 (14.3%) | 2 (14.3%) |
| **Welche drei der folgenden Punkte sind Ihrer Meinung nach am problematischsten hinsichtlich der Verwendung von Sprachmodellen in ihrem Fachgebiet? (geben Sie nur Ihre Top 3 an, wobei "1" = größter Nachteil) [Bedenken hinsichtlich der Datensicherheit und des Datenschutzes]** | **In your opinion, which three of the following are most problematic regarding the use of language models in your field? (indicate only your top 3, where "1" = biggest disadvantage) [Concerns about data security and data protection]** | **NaN** | **NaN** | 4 (11.1%) | 3 (4.3%) | 0 (0%) |
| | | **Nicht in Top 3** | **Not in top 3** | 12 (33.3%) | 30 (42.9%) | 8 (57.1%) |
| | | **1** | **1** | 11 (30.6%) | 19 (27.1%) | 5 (35.7%) |
| | | **2** | **2** | 6 (16.7%) | 14 (20%) | 1 (7.1%) |
| | | **3** | **3** | 3 (8.3%) | 3 (4.3%) | 0 (0%) |
| | | **Nichr in Top 3** | **Not in top 3** | 0 (0%) | 1 (1.4%) | 0 (0%) |
| **Welche drei der folgenden Punkte sind Ihrer Meinung nach am problematischsten hinsichtlich der Verwendung von Sprachmodellen in ihrem Fachgebiet? (geben Sie nur Ihre Top 3 an, wobei "1" = größter Nachteil) [Bedenken hinsichtlich der ärztlichen** | **In your opinion, which three of the following are most problematic regarding the use of language models in your specialty? (indicate only your top 3, where "1" = biggest disadvantage) [concerns about medical liability due to machine errors]** | **NaN** | **NaN** | 1 (2.8%) | 1 (1.4%) | 0 (0%) |
| | | **Nicht in Top 3** | **Not in top 3** | 14 (38.9%) | 34 (48.6%) | 7 (50%) |
| | | **1** | **1** | 6 (16.7%) | 10 (14.3%) | 1 (7.1%) |
| | | **2** | **2** | 7 (19.4%) | 15 (21.4%) | 3 (21.4%) |
| | | **3** | **3** | 8 (22.2%) | 10 (14.3%) | 3 (21.4%) |

| | | | | | | |
|---|---|---|---|---|---|---|
| **Haftung aufgrund von Maschinenfehlern]** | | | | | | |
| **Welche drei der folgenden Punkte sind Ihrer Meinung nach am problematischsten hinsichtlich der Verwendung von Sprachmodellen in ihrem Fachgebiet? (geben Sie nur Ihre Top 3 an, wobei "1" = größter Nachteil) [Mangelndes Vertrauen in "Black-Box" (intransparente) Diagnosen]** | **In your opinion, which three of the following are most problematic regarding the use of language models in your specialty? (indicate only your top 3, where "1" = biggest disadvantage) [Lack of trust in "black box" (non-transparent) diagnoses]** | **NaN** | **NaN** | 2 (5.6%) | 1 (1.4%) | 0 (0%) |
| | | **Nicht in Top 3** | **Not in top 3** | 8 (22.2%) | 21 (30%) | 5 (35.7%) |
| | | **1** | **1** | 10 (27.8%) | 17 (24.3%) | 2 (14.3%) |
| | | **2** | **2** | 9 (25%) | 17 (24.3%) | 3 (21.4%) |
| | | **3** | **3** | 7 (19.4%) | 14 (20%) | 4 (28.6%) |
| **Welche drei der folgenden Punkte sind Ihrer Meinung nach am problematischsten hinsichtlich der Verwendung von Sprachmodellen in ihrem Fachgebiet? (geben Sie nur Ihre Top 3 an, wobei "1" = größter Nachteil) [Diagnose und Behandlungsberatung erfolgen immer weniger durch dafür ausgebildete Fachärzte]** | **In your opinion, which three of the following are the most problematic with regard to the use of language models in your specialty? (indicate only your top 3, where "1" = biggest disadvantage) [Diagnosis and treatment advice are being given less and less by trained specialists]** | **NaN** | **NaN** | 5 (13.9%) | 2 (2.9%) | 0 (0%) |
| | | **Nicht in Top 3** | **Not in top 3** | 17 (47.2%) | 31 (44.3%) | 7 (50%) |
| | | **1** | **1** | 3 (8.3%) | 10 (14.3%) | 4 (28.6%) |
| | | **2** | **2** | 6 (16.7%) | 12 (17.1%) | 2 (14.3%) |
| | | **3** | **3** | 5 (13.9%) | 15 (21.4%) | 1 (7.1%) |
| **Welche drei der folgenden Punkte sind Ihrer Meinung nach am problematischsten hinsichtlich der Verwendung von Sprachmodellen in ihrem Fachgebiet? (geben Sie nur Ihre Top 3 an, wobei "1" = größter Nachteil) [Herausforderungen für die vertrauensvolle Beziehung zwischen Patient und Arzt]** | **In your opinion, which three of the following are the most problematic issues regarding the use of language models in your specialty? (indicate only your top 3, where "1" = biggest disadvantage) [Challenges for the trusting relationship between patient and doctor]** | **NaN** | **NaN** | 5 (13.9%) | 4 (5.7%) | 0 (0%) |
| | | **Nicht in Top 3** | **Not in top 3** | 16 (44.4%) | 27 (38.6%) | 6 (42.9%) |
| | | **1** | **1** | 5 (13.9%) | 12 (17.1%) | 3 (21.4%) |
| | | **2** | **2** | 4 (11.1%) | 14 (20%) | 3 (21.4%) |
| | | **3** | **3** | 6 (16.7%) | 13 (18.6%) | 2 (14.3%) |
| **Welche drei der folgenden Punkte sind Ihrer Meinung nach am problematischsten hinsichtlich der Verwendung von Sprachmodellen in ihrem Fachgebiet? (geben Sie nur Ihre Top 3 an, wobei "1" = größter Nachteil) [Bedenken hinsichtlich des Vergleichs und "Wettkampfes" von Ärzten mit Maschinen]** | **Which three of the following do you think are most problematic regarding the use of language models in your specialty? (indicate only your top 3, where "1" = biggest disadvantage) [Concerns regarding the comparison and "competition" of doctors with machines]** | **NaN** | **NaN** | 4 (11.1%) | 3 (4.3%) | 0 (0%) |
| | | **Nicht in Top 3** | **Not in top 3** | 22 (61.1%) | 45 (64.3%) | 9 (64.3%) |
| | | **1** | **1** | 1 (2.8%) | 5 (7.1%) | 2 (14.3%) |
| | | **2** | **2** | 5 (13.9%) | 6 (8.6%) | 2 (14.3%) |
| | | **3** | **3** | 4 (11.1%) | 11 (15.7%) | 1 (7.1%) |
| **Welche drei der folgenden Punkte sind Ihrer Meinung nach am problematischsten hinsichtlich der Verwendung von Sprachmodellen in ihrem Fachgebiet? (geben Sie nur Ihre Top 3 an, wobei "1" = größter Nachteil) [Auswirkungen auf den Personalbedarf]** | **In your opinion, which three of the following are most problematic regarding the use of language models in your field? (indicate only your top 3, where "1" = biggest disadvantage) [Impact on staffing needs]** | **NaN** | **NaN** | 5 (13.9%) | 4 (5.7%) | 0 (0%) |
| | | **Nicht in Top 3** | **Not in top 3** | 25 (69.4%) | 55 (78.6%) | 4 (28.6%) |
| | | **1** | **1** | 0 (0%) | 5 (7.1%) | 3 (21.4%) |
| | | **2** | **2** | 5 (13.9%) | 1 (1.4%) | 5 (35.7%) |
| | | **3** | **3** | 1 (2.8%) | 5 (7.1%) | 2 (14.3%) |
| | | **NaN** | **NaN** | 6 (16.7%) | 7 (10%) | 0 (0%) |

| | | | | | | |
|---|---|---|---|---|---|---|
| **Welche drei der folgenden Punkte sind Ihrer Meinung nach am problematischsten hinsichtlich der Verwendung von Sprachmodellen in ihrem Fachgebiet? (geben Sie nur Ihre Top 3 an, wobei "1" = größter Nachteil) [Andere]** | **In your opinion, which three of the following are the most problematic with regard to the use of language models in your field? (indicate only your top 3, where "1" = biggest disadvantage) [Other]** | **Nicht in Top 3** | **Not in top 3** | 26 (72.2%) | 59 (84.3%) | 11 (78.6%) |
| | | **1** | **1** | 1 (2.8%) | 1 (1.4%) | 1 (7.1%) |
| | | **2** | **2** | 1 (2.8%) | 1 (1.4%) | 2 (14.3%) |
| | | **3** | **3** | 2 (5.6%) | 2 (2.9%) | 0 (0%) |
| **Welche Berufsgruppe innerhalb Ihres Fachgebietes wird Ihrer Meinung nach am stärksten von der Einführung von Sprachmodellen betroffen sein (z.B. Pflegepersonal, Verwaltungspersonal, Ärzte/Ärztinnen, anderes Personal)** | **Which professional group within your specialty do you think will be most affected by the introduction of language models (e.g., nursing staff, administrative staff, doctors/physicians, other staff)** | *(free-text answers)* | | | | |
| **Inwieweit stimmen Sie der folgenden Aussage zu: “Mein Fachgebiet wird sich durch die Einführung von Sprachmodellen verbessern“? (1: Stimme überhaupt nicht zu, 5: Stimme voll und ganz zu)** | **To what extent do you agree with the following statement: "My subject area will improve as a result of the introduction of language models"? (1: Strongly disagree, 5: Strongly agree)** | **1.0** | **1.0** | 2 (5.6%) | 3 (4.3%) | 1 (7.1%) |
| | | **2.0** | **2.0** | 3 (8.3%) | 10 (14.3%) | 0 (0%) |
| | | **3.0** | **3.0** | 11 (30.6%) | 16 (22.9%) | 6 (42.9%) |
| | | **4.0** | **4.0** | 11 (30.6%) | 26 (37.1%) | 6 (42.9%) |
| | | **5.0** | **5.0** | 9 (25%) | 15 (21.4%) | 1 (7.1%) |
| **Wie würden Ihrer Meinung nach die meisten Fachleute innerhalb Ihres Fachgebietes die vorherige Frage im Vergleich zu Ihnen beantworten?** | **In your opinion, how would most experts in your field answer the previous question compared to you?** | **Sie würden im Durchschnitt eine ähnliche Antwort geben** | **On average, they would give a similar answer** | 14 (38.9%) | 32 (45.7%) | 5 (35.7%) |
| | | **Sie würden im Durchschnitt stärker widersprechen** | **On average, they would disagree more strongly** | 18 (50%) | 27 (38.6%) | 7 (50%) |
| | | **Sie würden im Durchschnitt stärker zustimmen** | **On average, they would agree more strongly** | 4 (11.1%) | 11 (15.7%) | 2 (14.3%) |

**Supplementary Table 2. Overview of the most common use cases that the participants mentioned for which they already use language models in their daily work**

| ID | Use Case | Mentions |
|---|---|---|
| U1 | Research tool | 17 |
| U2 | Question answering | 14 |
| U3 | Translation | 11 |
| U4 | Explanation of concepts | 11 |
| U5 | General text generation | 8 |
| U6 | E-mail drafting | 7 |
| U7 | Long text summarization | 6 |
| U8 | Studying support | 5 |
| U9 | Web search | 5 |
| U10 | Admission notes (*Arztbriefe*) | 5 |
| U11 | Programming | 4 |
| U12 | Spellcheck | 4 |

**Supplementary Table 3. Overview of the five most common key factors that respondents mentioned for improving the preparedness of their institutions for AI adoption with accompanying example quotes used to group them.**

| ID | Key Factor | # | Quotes (German, original) | Quotes (English, translated) |
|---|---|---|---|---|
| F1 | **Education about AI** | **46** | “Anwendung von Sprachmodellen auch ins medizinische Curriculum integrieren, dann fällt die Anwendung später leichter“<br>“Experten benennen und ausbilden, die dann wiederum Schulungen anbieten können”<br>“Sinnvolle Nutzung in die lehre inkorporieren – z.B. Seminare oder Praktika” | “Integrate the use of language models into the medical curriculum makes it easier to apply them later on“<br>“Appoint and train experts who can then offer training courses”<br>“Incorporate meaningful use into teaching - e.g. seminars or practical courses” |
| F2 | **Ensure Privacy & Security** | **17** | “Frühzeitige Einrichtung einer datensicheren Benutzerschnittstelle für das PKIS“<br>“Urheberrecht und Datenschutz klären. Aufklärung der Mitarbeiter über Potential und Fallstricke“<br>“Frage der Haftung, Sicherheit/ Fehleranfälligkeit der Systeme” | “Early establishment of a data-secure user interface for the employees“<br>“Clarify copyright and data protection. Educate employees about the potential and pitfalls“<br>“Question of liability, security/error-proneness of the systems” |
| F3 | **Infrastructure & Resources** | **13** | “Für ausreichend Hardware sorgen<br>“Schnittstellen von lokal betrieben LLMs zu den Patientenverwaltungssystemen.“<br>“Serverkapazitäten, Bereitschaft Technik zu beschaffen bzw. zu ersetzen um sinnvolle Einsatzmöglichkeiten zu erschließen” | “Ensure sufficient hardware<br>”Interfaces from locally operated LLMs to the patient administration systems."<br>“Server capacities, willingness to procure or replace technology in order to open up meaningful application possibilities” |
| F4 | **Provide Examples, Onboarding** | **10** | “Sich besser über die Möglichkeiten informieren und versuchen mit der Nutzung zu beginnen”<br>“In Vorlesungen und Seminare von Dozenten gezeigt bekommen, wie man Sprachmodellen vernünftig anwendet (praktische Beispiele im Alltag und in der Patienteninteraktion)“<br>“Anwendungen in den klinischen Alltag zeigen“ | “Be better informed about the possibilities and try to start using them“<br>“Be shown in lectures and seminars by lecturers how to use language models sensibly (practical examples in everyday life and in patient interaction)“<br>“Show applications in everyday clinical practice“ |
| F5 | **Digitalization of Clinical Texts** | **8** | “Frühzeitige Organisation einer Datenschutzstruktur, Digitalisierung von Befunden, Digitalisierung von Dialysedaten”<br>“Bessere einheitlichere Digitalisierung der Patientendaten und Möglichkeit der Kopplung dieser mit Sprachmodellen”<br>“Digitalisierung des deutschen Gesundheitswesen.... (keine Papierkurven mehr alles auf digitaler Form)“ | “Early organization of a data protection structure, digitization of findings, digitization of dialysis data“<br>“Better standardized digitization of patient data and possibility of linking this with language models”<br>“Digitization of the German healthcare system.... (no more paper charts, everything in digital form)” |
